# Estimating vaccine efficacy during open-label follow-up of COVID-19 vaccine trials based on population-level surveillance data

**DOI:** 10.1016/j.epidem.2024.100768

**Published:** 2024-04-15

**Authors:** Mia Moore, Yifan Zhu, Ian Hirsch, Tom White, Robert C. Reiner, Ryan M. Barber, David Pigott, James K. Collins, Serena Santoni, Magdalena E. Sobieszczyk, Holly Janes

**Affiliations:** a Fred Hutchinson Cancer Center, 1100 Fairview Ave N, Seattle, WA 98109, USA; b Sanofi, USA; c Biometrics, Vaccines, & Immune Therapies, BioPharmaceuticals R&D, AstraZeneca, Cambridge, UK; d Institute for Health Metrics and Evaluation within the Schools of Medicine at the University of Washington, Seattle, WA, USA; e Division of Infectious Diseases, Department of Medicine, Vagelos College of Physicians and Surgeons, New York-Presbyterian/Columbia University Irving Medical Center, New York, NY, USA

**Keywords:** Clinical trial design, Randomized controlled trial, Vaccine, Counterfactual

## Abstract

While rapid development and roll out of COVID-19 vaccines is necessary in a pandemic, the process limits the ability of clinical trials to assess longer-term vaccine efficacy. We leveraged COVID-19 surveillance data in the U.S. to evaluate vaccine efficacy in U.S. Government-funded COVID-19 vaccine efficacy trials with a three-step estimation process. First, we used a compartmental epidemiological model informed by county-level surveillance data, a “population model”, to estimate SARS-CoV-2 incidence among the unvaccinated. Second, a “cohort model” was used to adjust the population SARS-CoV-2 incidence to the vaccine trial cohort, taking into account individual participant characteristics and the difference between SARS-CoV-2 infection and COVID-19 disease. Third, we fit a regression model estimating the offset between the cohort-model-based COVID-19 incidence in the unvaccinated with the placebo-group COVID-19 incidence in the trial during blinded follow-up. Counterfactual placebo COVID-19 incidence was estimated during open-label follow-up by adjusting the cohort-model-based incidence rate by the estimated offset. Vaccine efficacy during open-label follow-up was estimated by contrasting the vaccine group COVID-19 incidence with the counterfactual placebo COVID-19 incidence. We documented good performance of the methodology in a simulation study. We also applied the methodology to estimate vaccine efficacy for the two-dose AZD1222 COVID-19 vaccine using data from the phase 3 U.S. trial (ClinicalTrials.gov # NCT04516746). We estimated AZD1222 vaccine efficacy of 59.1% (95% uncertainty interval (UI): 40.4%–74.3%) in April, 2021 (mean 106 days post-second dose), which reduced to 35.7% (95% UI: 15.0%–51.7%) in July, 2021 (mean 198 days post-second-dose). We developed and evaluated a methodology for estimating longer-term vaccine efficacy. This methodology could be applied to estimating counterfactual placebo incidence for future placebo-controlled vaccine efficacy trials of emerging pathogens with early termination of blinded follow-up, to active-controlled or uncontrolled COVID-19 vaccine efficacy trials, and to other clinical endpoints influenced by vaccination.

## 1. Introduction

The rapid development and evaluation of COVID-19 vaccines – and their remarkable efficacy – is one of the great success stories of the COVID-19 pandemic. As of April 17, 2023, a total of 11 vaccines were proven effective at preventing symptomatic COVID-19 (termed COVID-19 throughout), with higher efficacy against severe COVID-19, hospitalization, and death, and approved for use by either the United States (US) Food and Drug Administration (FDA) or the World Health Organization (WHO) (WH Organization, 2022; US Food and D Administration, 2022b). Streamlining the clinical development pathway was necessary, with the typical 7–10 year timeline for most vaccines shrunk to less than a year (Ball, 2020; Lance, 2021). This streamlining meant that efficacy trial results were reported to the field and vaccination offered to all trial participants very soon after efficacy was established, typically with two months of follow-up post-last vaccination, which was the level of safety follow-up required for FDA Emergency Use Authorization (EUA) (US Food and D Administration, 2022a). While necessary and appropriate given the pandemic context, rapid rollout of vaccines to trial participants meant that the ability to assess longer-term vaccine safety and efficacy in a controlled fashion were impacted: the trials ‘lost’ their placebo arms at the initiation of the open-label follow-up period. In particular, evaluating how vaccine efficacy (VE) varies over time, i.e., durability of vaccine efficacy, became considerably more challenging.

In the US, population-level surveillance data of the COVID-19 pandemic are reported by local or state public health authorities and include daily counts of diagnosed SARS-CoV-2 infections, COVID-19 hospitalizations, and deaths due to COVID-19 (Johns Hopkins Coronavirus Resource Center, 2020), although precisely what is reported varies considerably across regions and over time within a region. Population modeling based on these data impacted previous pandemic policies such as school and business closures, masking, vaccination and boosting (COVID-19 Scenario Modeling Hub, 2020; McBryde et al., 2020; Borchering et al., 2021; Abueg et al., 2021; Brauner et al., 2021). Specifically, modeling was utilized to estimate the spread of COVID-19 (Bertozzi et al., 2020), characterize the geographic regions and demographic sub-populations most impacted by the pandemic (Chitwood et al., 2022), and infer the effectiveness of non-pharmaceutical prevention interventions (Brauner et al., 2021; Duffey and Zio, 2020; Hasan et al., 2022; Panovska-Griffiths et al., 2021). Modeling was also proposed to determine the appropriate dosing regimen of COVID-19 vaccination and how dosing should evolve as the pandemic progressed (Giorgi et al., 2021; Selvaggio et al., 2021; Desikan et al., 2022).

Modeling has seldom been used to evaluate durability of COVID-19 vaccination efficacy. Yet, given that population-level COVID-19 surveillance data and models are rich in time and space, they may be applied to constructing ‘counterfactual’ placebo arms for COVID-19 vaccine trials for time periods after which placebo recipients have been vaccinated, for evaluating durability of vaccine efficacy (i.e., longer-term VE). There are many ways, however, in which the population-level surveillance data may not be comparable to COVID-19 vaccine trial endpoint data, including differences in trial populations vs. surveillance populations, capture of COVID-19 disease events, and population rollout of COVID-19 vaccines. Therefore, we combine these data with a U.S.-Government (USG)-funded COVID-19 vaccine trial (Lora et al., 2023), and develop methodology to estimate longer-term VE against COVID-19 disease by inferring counterfactual placebo COVID-19 incidence for vaccine trials conducted in the US. A critical and novel element of our methodology is that we leverage observed placebo COVID-19 incidence data for the initial placebo-controlled, blinded period of the trial to evaluate the ‘offset’: the difference between the population-based incidence and the placebo-arm incidence. Our estimate of counterfactual placebo incidence for the open-label follow-up trial period is then adjusted by this estimated offset. This correction is only possible in the context of a study with parallel blinded placebo follow-up and population surveillance data. It is valid under the assumption that the offset, allowed to depend on covariates, is constant in calendar time, and applies to SARS-CoV-2 variants circulating over both blinded and open-label periods of the trial. A second critical feature of our methodology is the integration of multiple sources of uncertainty.

An outline of the paper is as follows. In Section 2, we describe methodology to estimate VE during the open-label follow-up period of a placebo-controlled trial to assess vaccine efficacy durability. Sections 3.1 and 3.2 illustrate a simulation study we conducted to evaluate performance of the proposed methodology in scenarios designed to reflect the USG-funded COVID-19 vaccine trials. Section 3.3 applies the methodology to evaluating longer-term VE of the AZD1222 vaccine in the USG-funded phase 3 trial conducted in the U.S. and South America (Falsey et al., 2021; Sobieszczyk et al., 2022). We end with a discussion of potential applications and future work.

## 2. Methods

### 2.1. Setting

Our focus is the setting of a randomized, blinded COVID-19 VE trial conducted at multiple clinical trial sites. Details of the prototypical trial design used by USG-funded COVID-19 VE trials are described elsewhere (Lora et al., 2023; Falsey et al., 2021; Baden et al., 2021; Sadoff et al., 2021; Dunkle et al., 2022; Mehrotra et al., 2021; Follmann et al., 2021). Briefly, adults at appreciable risk of SARS-CoV-2 infection or COVID-19 were enrolled and randomized to receive vaccine or placebo with 1:1 or 2:1 randomization ratios. Individuals with known prior SARS-CoV-2 infection were not eligible. Immediately following enrollment and randomization, participants underwent a *baseline* assessment during which demographic information, behavioral characteristics and medical history were recorded, referred to from here as their baseline covariates. During the baseline assessment, participants were also tested for evidence of infection prior to enrollment. Given the delays in testing, participants with positive test were still enrolled in the study and received study product, although these individuals were excluded from estimates of vaccine efficacy. This subset is assumed to be similar across arms due to randomization. Participants were followed for the primary endpoint, polymerase chain reaction (PCR)-confirmed COVID-19. At a given point in calendar time, often after interim analyses demonstrating efficacy of the vaccine, trial participants were unblinded to randomization assignment and offered study vaccine, or elected to procure vaccine outside of the study through community providers. We refer to the transition from blinded to open-label follow-up as the study *crossover time*. After the crossover time, participants continued to be followed for COVID-19 in an open-label fashion until all participants reached the end of trial follow-up, typically one or two years post-randomization.

A key secondary objective of each study was to evaluate how VE against COVID-19 varied over time, i.e., durability of VE. Primary interest was evaluating time-varying VE in the *primary efficacy cohort* of the trials, typically defined as the subset of SARS-CoV-2 negative participants at enrollment; this subgroup had the most potential to benefit from vaccination. Also, most trials considered primary endpoint COVID-19 as those occurring 14 or more days post-last vaccination to allow vaccine-induced immune responses to mount. Accordingly, we focused on evaluating time-varying VE in the primary efficacy cohort and counting only primary endpoint COVID-19.

#### 2.1.1. COVID-19 terminology

Throughout this manuscript we refer to COVID-19, SARS-CoV-2 infection, and SARS-CoV-2 positive tests. To help orient the reader we will include a quick summary of how each is used and when.

- **SARS-CoV-2 infection:** All viral infections regardless of symptom status and diagnosis. These events are tracked internally within our models but are not always observed.
- **COVID-19:** The subset of SARS-CoV-2 infections that lead to symptomatic COVID-19 disease. We define vaccine efficacy as the decrease in the number of such outcomes.
- **SARS-CoV-2 test-positivity:** A positive result, at the time of study enrollment for either (1) a nucleic acid amplification test (NAAT) that detects ongoing infection, or (2) an antibody test to detect prior infection. Individuals who are positive on either test are excluded from estimates of vaccine efficacy, however we use the rates of positive tests in model calibration.

### 2.2. Target parameter

Let $Z_{i}$ denote randomization assignment for participant i, where $Z_{i}=1$ denotes vaccine and $Z_{i}=0$ denotes placebo. Let $S_{i}$ denote the study site at which a participant is recruited, and $V_{i}$ denote a vector of baseline covariates including age, race and ethnicity, pre-existing conditions related to severe COVID-19 disease, and self-reported SARS-CoV-2 infection exposure risk factors. Both $V_{i}$ and $S_{i}$ are used to model population incidence of SARS-CoV-2 infection and COVID-19. A subset of baseline covariates $V_{i}$, denoted by $X_{i}$, are considered in estimating incidence in the trial. $X_{i}$ includes participant age, race and ethnicity, and geographic region which are potentially associated with SARS-CoV-2 infection, exposure, and censoring. Trial participants randomized to placebo in the primary efficacy cohort are followed for primary endpoint COVID-19 through $L_{i}$, the earliest of unblinding, outside COVID-19 vaccination, or study termination, called *blinded primary follow-up* with *unblinding* censoring. For placebo-group participants, trial follow-up after unblinding continues but is not utilized in our analysis. Participants randomized to vaccine are followed over both blinded primary follow-up and follow-up during the open-label period through to the earliest of outside COVID-19 vaccination, study termination or end of study follow-up, called *all follow-up* with *end of study* censoring. Consistent with others (Follmann and Fay, 2022; Fintzi and Follmann, 2021; Lin et al., 2021), we use a calendar time scale for analysis given the dramatic temporal variation in COVID-19 incidence. Let $Y_{i}(m)$ be the indicator of observing a primary endpoint event for participant i during calendar month m. Let $R_{i}(m)$ denote the number of days at risk for participant i during month m, i.e. the number of days during which the participant was followed by the trial and had not yet experienced a study endpoint. The total number of events and total amount of person-time, measured in person-days, for month m, randomization arm z, and covariate stratum x are denoted by $Y_{z}^{x}(m)$ and $R_{z}^{x}(m)$, respectively. For the placebo group, we have observations $\left(Y_{0}^{x}(m),R_{0}^{x}(m)\right)$ for $m=1,\ldots ,M_{u}$, where m=1 is the first month with blinded follow-up accruing in the placebo group and $M_{u}$ is the last calendar month before the crossover time. For the vaccine group, we have observations $\left(Y_{1}^{x}(m),R_{1}^{x}(m)\right)$ for $m=1,\ldots ,M_{f}>M_{u}$, where $M_{f}$ is the last calendar month with follow-up through end-of-study censoring. We target estimation of marginal COVID-19 incidence in arm z during the open-label period, defined as

(1) $$\Lambda_{z}\left(m\right)=\sum_{x}\Lambda_{z}^{x}\left(m\right)w_{z}^{x},$$

for $m=M_{u}+1,\ldots ,M_{f}$, where $\Lambda_{z}^{x}(m)$ is the COVID-19 incidence in covariate stratum x of arm z and month m, and $w_{z}^{x}$ is the proportion of trial participants enrolled in arm z who are in stratum x. This marginal incidence parameter allows covariates X to impact SARSCoV-2 exposure and/or early unblinding or study termination. While $\Lambda_{1}(m)$ can be estimated directly from trial data using $\left(Y_{1}^{x}(m),R_{1}^{x}(m)\right)$ for $m=M_{u}+1,\ldots ,M_{f}$, $\Lambda_{0}(m)$ is not directly estimable using trial data for $m>M_{u}$ and its estimation is the topic of this paper.

We target estimation of VE during the open-label period, defined as

(2) $$VE\left(m\right)=1-\frac{\Lambda_{1}\left(m\right)}{\Lambda_{0}\left(m\right)},$$

$$m=M_{u}+1,\ldots ,M_{f}.$$

In practice, unblinding and crossover of trial participants may take weeks or months, given that participants typically need to come into the clinic to learn their randomization assignment. In our applications, we select $M_{u}$ as the last calendar month at which at least 50% of placebo participants contribute blinded follow-up. We explore the impact of this choice in Supplementary Materials Section S1.1.

Throughout the manuscript, $Y_{z}^{x}(m)$, $R_{z}^{x}(m)$, $\Lambda_{z}^{x}$ and $VE_{z}^{x}$ refer to true parameter values. Parameters with hat superscripts refer to estimated values, and parameters with tilde superscripts refer to intermediate estimates based on the cohort model.

### 2.3. Methodology

**Fig. 1.**
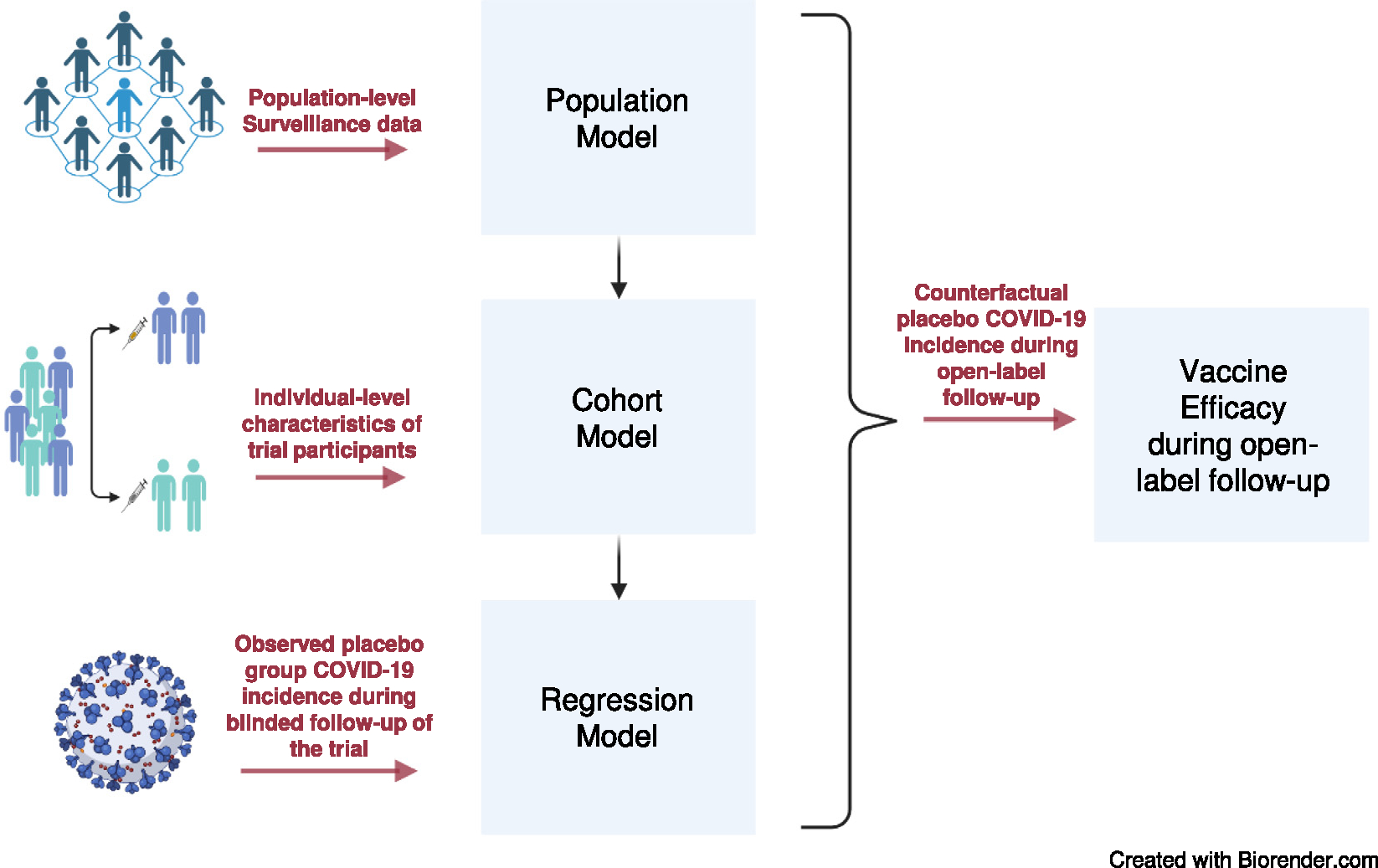
Schematic of the approach to estimating counterfactual placebo COVID-19 incidence.

Fig. 1 shows a schematic of our methodology, which is described in detail in the sections that follow. Briefly, county population-level surveillance data from the U.S. on diagnosed SARS-CoV-2 infections, COVID-19 hospitalizations, deaths due to COVID-19, SARS-CoV-2 sero-prevalence studies, and a mechanistic model with parameters based on published literature are used as the basis for a *population model* that characterizes SARS-CoV-2 infection incidence among the unvaccinated. Next, the population-model-based county-level SARS-CoV-2 infection incidence in the unvaccinated is the input to a *cohort model* that adjusts the population model to the trial cohort and takes into account the difference between SARS-CoV-2 infection and COVID-19. The cohort model is a mechanistic model that takes into account trial eligibility criteria and individual-level characteristics of the trial participants including baseline SARS-CoV-2 status. A *regression model* is then used to contrast the cohort-model-based COVID-19 incidence in the unvaccinated with the observed placebo COVID-19 incidence in the trial during blinded follow-up, i.e., before the crossover time. This regression model estimates the *offset* which measures the difference between the cohort-model-based and observed placebo incidence rates. The counterfactual placebo COVID-19 incidence estimate is the cohort-model-based incidence rate adjusted by the estimated offset. After the crossover time, VE is estimated by contrasting the observed open-label vaccine group incidence with the counterfactual placebo incidence. Details of each of these steps follow.

### 2.4. Population model

**Fig. 2.**
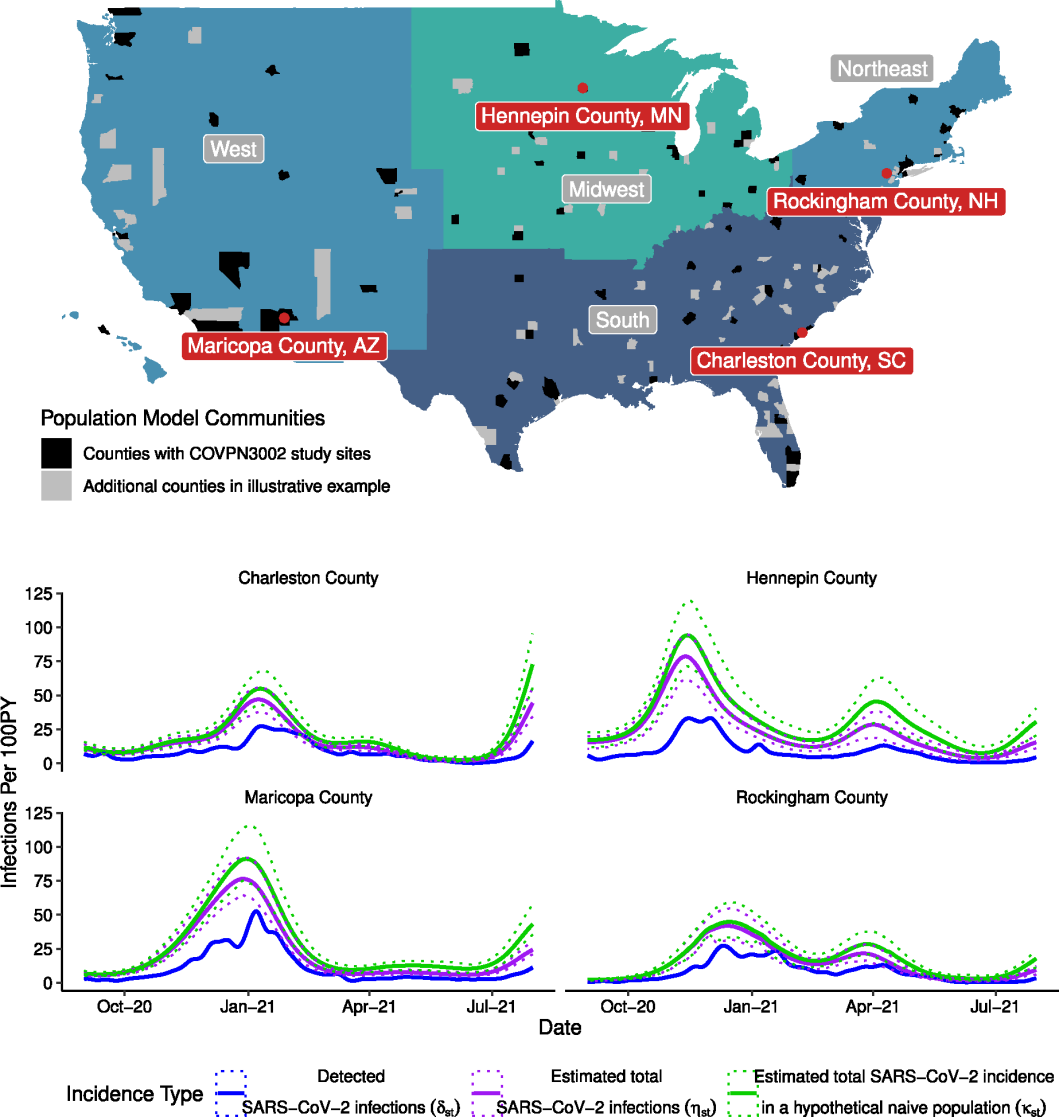
Population model summary. The population model provides estimates of incidence in each state and in dozens of counties. We highlight the estimated incidence in four counties from September 1, 2020 to July 31, 2021. The smoothed number of daily detected SARS-CoV-2 infections per-capita is shown in blue. The model outputs are the estimated number of SARS-CoV-2 infections per capita (both detected and undetected; purple) and the estimated number of SARS-CoV-2 infections per capita in a hypothetical naive population without prior SARS-CoV-2 infection or COVID-19 vaccination (green). (For interpretation of the references to color in this figure legend, the reader is referred to the web version of this article.)

We leverage an existing global population model for COVID-19 incidence built by the International Health Metrics and Evaluation (IHME) group at the University of Washington, described in detail elsewhere (Barber et al., 2022; Reiner et al., 2022). Briefly, the population model uses numbers of reported COVID-19- associated hospitalizations and deaths, and SARS-CoV-2 sero-prevalence surveys to estimate SARS-CoV-2 incidence over time at the state or county level for each study site. We illustrate the performance of the population model using incidence estimates from four counties hosting trial sites during the time period September, 2020–July, 2021. Even after smoothing to account for day of week and holiday effects, the number of detected SARS-CoV-2 infections at site s on day t including both asymptomatic infections and COVID-19 ($\delta_{st}$, Fig. 2, blue curve) still exhibits variability due to reporting lags (Badker et al., 2021; Simon, 2021). Crucially, $\delta_{st}$ will also underestimate the total number of infections by a potentially time variable factor dependent on testing availability and usage. The estimated total number of SARS-CoV-2 infections at site s on day t, $\eta_{st}$, which leverages hospitalization and death rates and state-level sero-prevalence, corrects for this factor (Fig. 2, purple curve). Finally, the population model also accounts for increasing population-level immunity due to prior infection and COVID-19 vaccination, and estimates SARS-CoV-2 incidence rate in a hypothetical naive representative subpopulation that has no vaccine-induced or infection-induced immunity on day, $\kappa_{st}$ (Fig. 2, green curve). This hypothetical subpopulation represents an idealized placebo cohort at the time of enrollment whose incidence depends only the current SARS-CoV-2 prevalence, contact rates, and per-contact infectiousness in the general population. Although a true placebo cohort would still accumulate immunity, it does so in a manner distinct from the general population, as described in the next section. The overall incidence and incidence in this hypothetical naive population begin to diverge in December 2020 when there is a significant wave of infections, and diverge further in spring 2021 when COVID-19 vaccines became widely available in the population. Failing to account for this population-level immunity is therefore important to prevent attenuation of placebo estimates over time (see Supplementary Materials Section S1.2).

### 2.5. Cohort model

Incidence of COVID-19 in the trial population is expected to differ from SARS-CoV-2 incidence in the general population due to the exclusion of asymptomatic infections, trial eligibility criteria and recruitment strategies, and individual self-selection for trial participation. We build a cohort model to adjust for differences in SARS-CoV-2 exposure and susceptibility to COVID-19 in the trial cohort as compared to the population at large.The cohort model depends only on information collected at baseline (i.e, during enrollment) and does not use any incidence data collected during either blinded or open-label follow-up. The purpose of the cohort model is to project COVID-19 incidence in the placebo arm in the absence of any vaccination.

We model each individual placebo recipient’s log-hazard ratio of SARS-CoV-2 infection relative to the local population, $\alpha_{i}$, and log-odds of COVID-19 given infection, $\rho_{i}$, using a set of generalized linear models (GLMs), with study site $\left(S_{i}\right)$ and behavioral and demographic characteristics $\left(V_{i}\right)$ as predictors.


(3) $$\begin{array}{ll} \mathrm{Subject}\;\mathrm{characteristics},\;\mathrm{site} & \left\{V_{i},S_{i}\right\}\\ \mathrm{Log}\;\mathrm{hazard}\;\mathrm{ratio}\;\mathrm{of}\;\mathrm{infection}\left(\mathrm{GLM}\;\mathrm{output}\right) & \log\;\alpha_{i}=\beta^{\alpha }V_{i}+\alpha_{S_{i}}\\ \mathrm{Site-level}\;\mathrm{random}\;\mathrm{effect} & \alpha_{S_{i}}∼N\left(\mu_{\alpha },\sigma_{\alpha }\right)\\ \mathrm{Log}\;\mathrm{odds}\;\mathrm{of}\;\mathrm{COVID-19}\;\mathrm{given} & \\ \mathrm{SARS-CoV-2}\;\mathrm{infection}\;\left(\mathrm{GLM}\;\mathrm{output}\right) & \log\frac{\rho_{i}}{1\;-\;\rho_{i}}=\beta^{\rho }V_{i} \end{array}$$

We assume that these individual-level adjustments, $\alpha_{i}$ and $\rho_{i}$ are constant in time. In our application, we calculate $\beta^{\alpha }V_{i}$ based on an exposure score (described in Supplementary Materials Section S1.3.1) and $\beta^{\rho }V_{i}$ using coefficients from Supplemental Table S4 (described in Supplemental Section S1.3.2). In addition, $\alpha_{i}$ is modified by a site-level random effect, $\alpha_{S_{i}}$, representing a site’s tendency to have recruited individuals at higher or lower risk of SARS-CoV-2 infection than the general population in ways that are not sufficiently captured by the covariates in V. These site-level effects are calibrated by comparing the observed SARS-CoV-2 positivity at enrollment for participant i (by PCR or serology), $B_{i}$, with their predicted model-based estimate of the probability of baseline SARS-CoV-2 positivity, $\xi_{i}$. See Supplementary Materials sections S1.3.3 for details on estimation of $\xi_{i}$ and S1.3.4 for details on calibration of $\alpha_{S_{i}}$ and hyperparameters $\mu_{\alpha }$ and $\sigma_{\alpha }$, respectively.

The study endpoint rate for individual i on day $d,\;y_{i,d}$, depends on (1) $\alpha_{i}$ and $\rho_{i}$ as defined above, (2) the population level SARS-CoV-2 incidence at their site, $\kappa_{S_{i},d}$, as estimated by the population model and (3) the probability that an individual remains uninfected up to day $d,\;P_{i,d}^{Unf}$. Some patients may have become infected prior to their enrollment date, $E_{i}$, and still tested negative during screening due to waning antibodies. To account for this we set the initial probability of remaining uninfected to $v_{i}$ (see Supplementary Material Section S1.3.3 for further details). In addition, asymptomatic SARS-CoV-2 infections during trial follow-up are not captured as COVID-19 endpoints. Individuals with an asymptomatic SARS-CoV-2 continue to accrue follow-up time, $r_{i,d}$, but cannot be reinfected.


(4) $$\begin{array}{ll} \mathrm{Probability}\;\mathrm{uninfected} & P_{i,d}^{\mathrm{Unf}}=\exp\left[-\gamma_{i,d}\right]P_{i,d-1}^{\mathrm{Unf}}\;P_{i,E_{i}}^{\mathrm{Unf}}=v_{i}\\ \text{Daily}\;\text{hazard}\;\text{rate}\;\text{of}\;\text{SARS-CoV-2}\;\text{infection} & \gamma_{i,d}=\frac{\alpha_{i}}{E^{S_{i}}[\alpha ]}\kappa_{S_{i},d}\\ \text{Daily}\;\text{probability}\;\text{of}\;\text{symptomatic}\;\text{COVID-19} & y_{i,d}=\rho_{i}\left(1-\exp\left[-\gamma_{i,d}\right]\right)P_{i,d-1}^{\mathrm{Unf}}\;d>E_{i}\\ \text{Probability}\;\text{accruing}\;\text{follow-up} & r_{i,d}=r_{i,d-1}-y_{i,d}\;r_{i,E_{i}}=1 \end{array}$$

**Fig. 3.**
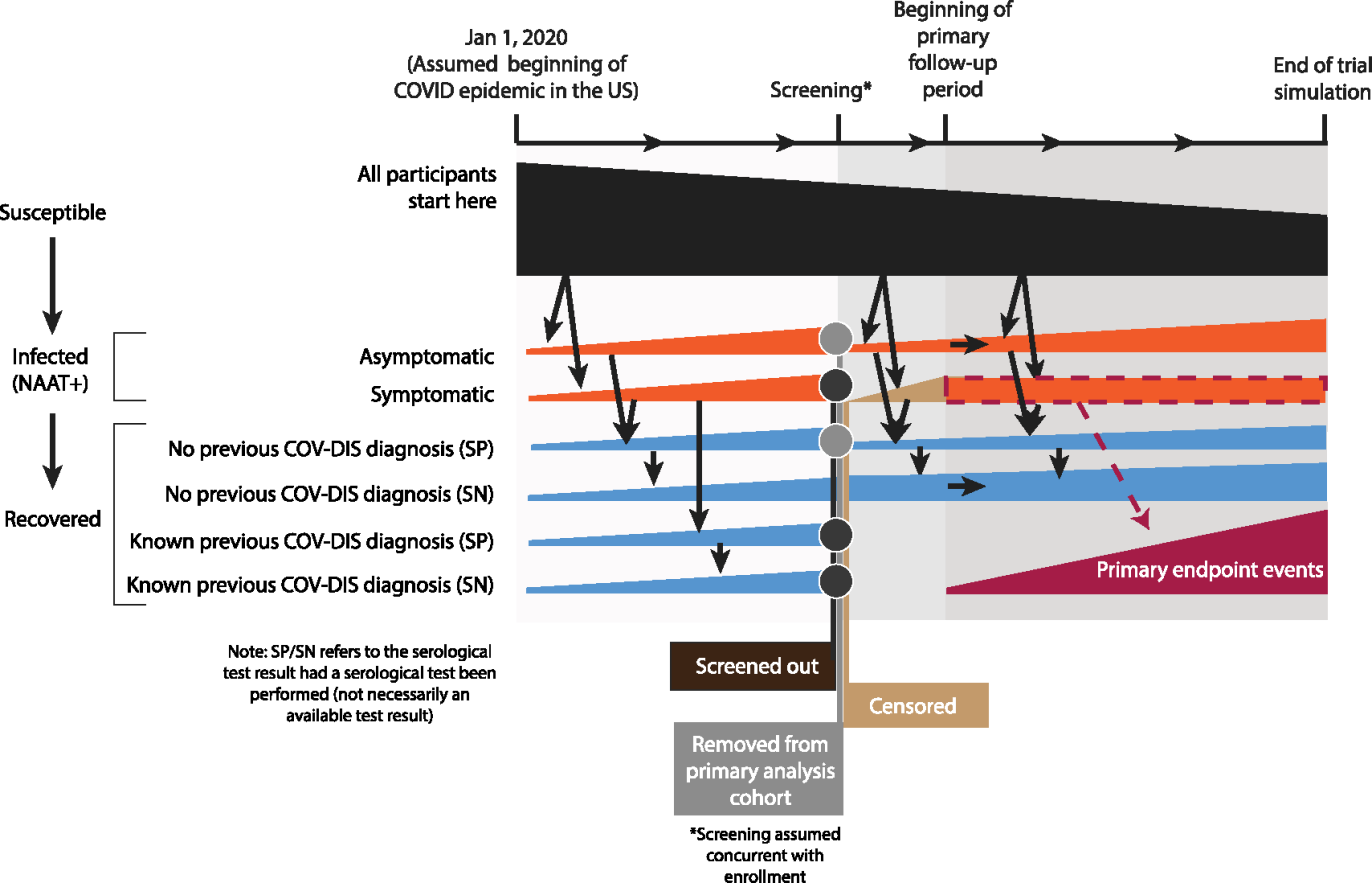
Conceptual framework of the cohort model. All individuals are assumed to be uninfected as of Jan 1, 2020. Based on the population model and individual characteristics, individuals become infected either symptomatically or asymptomatically. The proportion of symptomatic infections that are diagnosed depends on the study site, whereas all asymptomatic infections are assumed to be undetected. Infected individuals are initially NAAT+, then seropositive, then eventually seronegative. Anyone with known previous diagnosis or currently showing symptoms of COVID-19 (COV-DIS) is screened out of the trial, whereas those who are later found to be either NAAT+ or seropositive are excluded from the primary analysis cohort. The primary analysis cohort therefore consists primarily of uninfected individuals, but with some individuals with natural immunity. Starting at the beginning of the primary follow-up period, all symptomatic infections are counted as primary endpoints. Credit: Lindsay Carpp.

The expectation $E^{S_{i}}[\alpha ]$ refers to the site-specific, mean value of α in the general population. For simplicity, this model framework assumes no incubation period. The tracking of participant infection status and enrollment is summarized in Fig. 3.

Primary endpoints, i.e. those occurring on or after day $T_{i}$, typically 14 days afters the final dose, are aggregated by stratum X for each month $m=1,\ldots ,M_{f}$. The cohort model provides estimates of incidence both during primary blinded follow-up, denoted with the subscript a, and from the time of censoring, $L_{i}$, to study termination, denoted by the subscript p. Cohort-model-based estimates of the expected number of primary endpoints, ${\tilde{Y}}_{0,a}^{x}(m)$, and person-time, ${\tilde{R}}_{0,a}^{x}(m)$, before censoring are used to estimate the offset between the cohort model and observed trial incidence as described below in Section 2.6. Cohort-model-based estimates of the number of placebo group events, ${\tilde{Y}}_{0,p}^{x}(m)$, and person-time, ${\tilde{Y}}_{0,p}^{x}(m)$ after censoring are used to estimate counterfactual placebo incidence during open-label follow-up as described below in Section 2.7.


(5) $${\tilde{Y}}_{0,a}^{x}\left(m\right)=\sum_{i,d:\left\{m\left(d\right)=m,T_{i}\leq d\leq L_{i},X_{i}=x,Z_{i}=0\right\}}\left(1-B_{i}\right)y_{i,d}\;{\tilde{R}}_{0,a}^{x}\left(m\right)=\sum_{i,d:\left\{m\left(d\right)=m,T_{i}\leq d\leq L_{i},X_{i}=x,Z_{i}=0\right\}}\left(1-B_{i}\right)r_{i,d}\;{\tilde{Y}}_{0,p}^{x}\left(m\right)=\sum_{i,d:\left\{m\left(d\right)=m,d>L_{i},X_{i}=x,Z_{i}=0\right\}}\left(1-B_{i}\right)y_{i,d}\;{\tilde{R}}_{0,p}^{x}\left(m\right)=\sum_{i,d:\left\{m\left(d\right)=m,d>L_{i},X_{i}=x,Z_{i}=0\right\}}\left(1-B_{i}\right)r_{i,d}$$

In order to capture uncertainty in the model-based estimates, the cohort model is re-run $N_{\text{cohort}}$ times. In each run, a new realization for the population model is drawn at random, new values for the risk model coefficients $\beta^{\alpha }$ and $\beta^{\phi }$ are drawn from their distributions, and new site-level effects $\alpha_{S_{i}}$ are calibrated for all sites. Section 2.8 describes how these contribute to characterizing uncertainty in the counterfactual placebo incidence and VE estimates.

### 2.6. Regression model

We use Poisson regression to estimate the offset, i.e. the difference between the model-based COVID-19 incidence and observed placebo group COVID-19 incidence during blinded follow-up. Specifically, we estimate a contrast of the observed placebo group COVID-19 incidence, ${\overset{ˆ}{\Lambda }}_{0}^{x}(m)=\frac{Y_{0}^{x}(m)}{R_{0}^{x}(m)}$, and the model-based estimate of COVID-19 incidence pre-unblinding, ${\tilde{\Lambda }}_{0,a}^{x}(m)=\frac{{\tilde{Y}}_{0,a}^{x}(m)}{R_{0,a}^{x}(m)}$ for $m=1,\ldots ,M_{f}$ and stratum X=x.

We allow the offset to depend on X to account for potentially variable model performance depending on geographic region and/or participant demographics. We focus on the log incidence ratio as the contrast function, i.e., $log\;\frac{{\overset{ˆ}{\Lambda }}_{0}^{x}(m)}{{\overset{ˆ}{\Lambda }}_{0,a}^{x}(m)}$. The Poisson regression model is as follows:

(6) $$\log E\left(Y_{0}^{X}\left(m\right)\right)=\log R_{0}^{X}\left(m\right)+\log{\tilde{\Lambda }}_{0,a}^{X}\left(m\right)+\mu X.$$

from which we can derive the estimated offset $\overset{ˆ}{\mu }X$.

Note that this analysis uses all blinded primary follow-up time in the placebo arm; placebo-arm participants contribute until the time of COVID-19 event or unblinding censoring. Due to the potential for informative unblinding (Tsiatis and Davidian, 2021), we also perform a sensitivity analysis in which all placebo-arm participants are censored after the crossover month $M_{u}$ (see Supplementary Materials Section S1.1).

Importantly, the offset, $\overset{ˆ}{\mu }X$, depends on X but is assumed to be constant in m. Therefore, the resultant estimated counterfactual placebo incidence estimate will be valid under the assumption that the offset is constant in calendar time, and in particular that the offset estimated for SARS-CoV-2 variants circulating prior to crossover applies to variants circulating post-crossover. The assumption of a time-constant offset over all m is not testable, although we can assess whether or not the offset remains constant during the blinded follow-up period (see Supplementary Materials Section S1.4).

We estimate counterfactual placebo COVID-19 incidence as

(7) $${\overset{ˆ}{\Lambda }}_{0}^{CF,x}(m)=\left\{\begin{array}{ll} {\tilde{\Lambda }}_{0,a}^{x}(m)e^{\overset{ˆ}{\mu }x}, & m\leq M_{u}\\ \frac{{\tilde{\Lambda }}_{0,a}^{x}(m){\tilde{R}}_{0,a}^{x}(m)+{\tilde{\Lambda }}_{0,p}^{x}(m){\tilde{R}}_{0,p}^{x}(m)}{{\tilde{R}}_{0,a}^{x}(m)+{\tilde{R}}_{0,p}^{x}(m)}e^{\overset{ˆ}{\mu }x}, & m>M_{u} \end{array}\right.$$

(8) $${\tilde{R}}_{0,a/p}^{x}=\sum_{i:X_{i}=x,Z_{i}=0}{\tilde{R}}_{i,a/p}$$

In other words, for $m\leq M_{u}$, the estimated counterfactual placebo COVID-19 incidence in stratum x and month m is calculated by applying the estimated x-specific offset to the model-based estimate of COVID-19 incidence pre-unblinding, ${\tilde{\Lambda }}_{0,a}^{x}(m)$. For $m>M_{u}$, the estimated offset is applied to a weighted average of the model-based COVID-19 incidence pre-unblinding, ${\tilde{\Lambda }}_{0,a}^{x}(m)$, and post-unblinding, ${\tilde{\Lambda }}_{0,p}^{x}(m)=\frac{{\tilde{Y}}_{0,p}^{x}(m)}{{\tilde{R}}_{0,p}^{x}(m)}$ weighted by the amount of actual and projected follow-up time.

### 2.7. Estimation of marginal incidence and VE

Given the observed COVID-19 incidence in stratum x, randomization group z, and month m, ${\overset{ˆ}{\Lambda }}_{Z}^{x}(m)=\frac{Y_{Z}^{x}(m)}{R_{Z}^{x}(m)}$, the marginal observed incidence in month m is estimated by

(9) $${\overset{ˆ}{\Lambda }}_{z}(m)=\frac{\sum_{x}\;{\overset{ˆ}{\Lambda }}_{z}^{x}(m){\overset{ˆ}{w}}_{z}^{x}(m)}{\sum_{x}\;{\overset{ˆ}{w}}_{z}^{x}(m)},$$

where ${\overset{ˆ}{w}}_{z}^{x}(m)$ is equal to the total number of participants enrolled in stratum x and arm z, unless $R_{z}^{x}(m)=0$ in which case ${\overset{ˆ}{w}}_{z}^{x}(m)=0$. This estimator is implemented for $m=1,\ldots ,M_{u}$ for Z=0, and for $m=1,\ldots ,M_{f}$ for Z=1.

Given the estimated counterfactual placebo incidence in stratum x and month m defined in (8), the marginal counterfactual placebo incidence in month m is estimated by

(10) $${\overset{ˆ}{\Lambda }}_{0}^{CF}(m)=\frac{\sum_{x}\;{\overset{ˆ}{\Lambda }}_{0}^{CF,x}(m){\overset{ˆ}{w}}_{0}^{x}(m)}{\sum_{x}\;{\overset{ˆ}{w}}_{0}^{x}(m)}.$$

For the blinded follow-up period prior to crossover, observed VE for month m is estimated as

(11) $${\hat{VE}}_{obs}\left(m\right)=1-\frac{{\overset{ˆ}{\Lambda }}_{1}\left(m\right)}{{\overset{ˆ}{\Lambda }}_{0}\left(m\right)},m=1,\ldots ,M_{u}.$$

Counterfactual VE for month m is estimated over all follow-up as

(12) $${\hat{VE}}_{CF}\left(m\right)=1-\frac{{\overset{ˆ}{\Lambda }}_{1}\left(m\right)}{{\overset{ˆ}{\Lambda }}_{0}^{CF}\left(m\right)},m=1,\ldots ,M_{f}.$$

### 2.8. Estimation of uncertainty intervals

We utilize a non-parametric bootstrap procedure to quantify uncertainty in the COVID-19 incidence and VE estimates. The procedure captures uncertainty in the cohort model, which is contributed by the population model and parameter uncertainty in the cohort model, statistical uncertainty in the offset estimated by the regression model, and variability due to trial participant characteristics. For this reason, we use the term *uncertainty intervals* because the intervals capture not only statistical uncertainty but uncertainty in non-identifiable parameters in the cohort model.

We bootstrap K=500 samples. For bootstrap iteration k,k=1…K, the procedure is as follows:

1. Sample one of the $N_{cohort}=100$ cohort model runs. Denote the outputs of this run by ${\tilde{Y}}_{0,a,k}^{X}(m)$, ${\tilde{R}}_{0,a,k}^{X}(m)$, ${\tilde{Y}}_{0,p,k}^{X}(m)$, ${\tilde{R}}_{0,p,k}^{X}(m)$, ${\tilde{\Lambda }}_{0,a,k}^{X}(m)={\tilde{Y}}_{0,a,k}^{X}(m)/{\tilde{R}}_{0,a,k}^{X}(m)$ and ${\tilde{\Lambda }}_{0,p,k}^{X}(m)={\tilde{Y}}_{0,p,k}^{X,k}(m)/{\tilde{R}}_{0,p,k,k}^{X}(m)$, $m=1,\ldots ,M_{f}$;
2. Resample participants from each arm Z and stratum X with replacement. Calculate observed event counts, $Y_{Z,k}^{X}(m)$, and person-time, $R_{Z,k}^{X}(m)$, where $m=1,\ldots ,M_{u}$ for Z=0 and $m=1,\ldots ,M_{f}$ for Z=1. Calculate observed incidence, ${\overset{ˆ}{\Lambda }}_{Z,k}^{X}(m)=Y_{Z,k}^{X}(m)/R_{Z,k}^{X}(m)$;
3. Fit the Poisson regression model to $\left(Y_{0,k}^{X}(m),R_{0,k}^{X}(m)\right)$ and ${\tilde{\Lambda }}_{0,a,k}^{X}(m)$, and estimate counterfactual incidence for stratum X, ${\overset{ˆ}{\Lambda }}_{0,k}^{CF,X}(m)$;
4. Estimate marginal incidence ${\overset{ˆ}{\Lambda }}_{Z,k}(m)$ for Z=0,1 and marginal counterfactual placebo incidence ${\overset{ˆ}{\Lambda }}_{0,k}^{CF}(m)$;
5. Estimate ${\hat{VE}}_{\text{obs},k}(m)$ and ${\hat{VE}}_{k}(m)$.

The (1–α) level uncertainty interval is defined as the α/2 and (1–α/2) percentiles of the K estimates.

## 3. Results

### 3.1. Simulation: Reliable estimation of VE

**Fig. 4.**
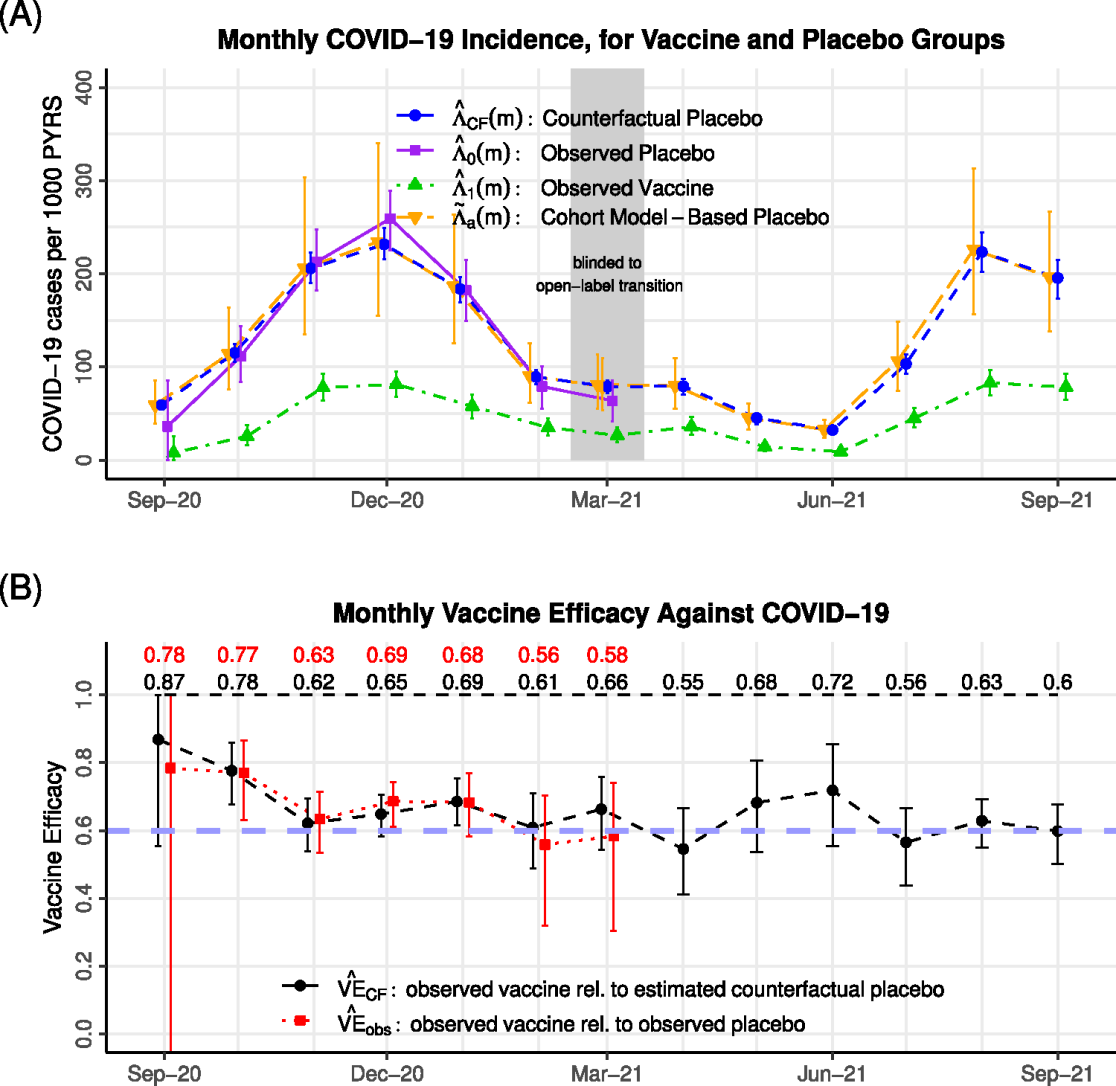
Estimated COVID-19 incidence for illustrative simulated dataset. (A) Observed vaccine and placebo group COVID-19 incidence is shown during blinded follow-up, and observed vaccine group incidence is shown during open-label follow-up. The cohort-model based estimate of incidence, and the estimate of counterfactual placebo incidence, is shown over all follow-up. (B) Estimated monthly VE, based on the contrast between observed vaccine vs. placebo group incidence during blinded follow-up (observed VE), and observed vaccine group vs. counterfactual placebo incidence during all follow-up (counterfactual VE).

We begin by illustrating the methodology using a simulated dataset that mimics features of USG-funded phase 3 COVID-19 VE trials, where the true level of vaccine efficacy is known. A total of 30,000 participants were enrolled in the illustrative trial, randomized in a 2:1 ratio to two doses of vaccine, administered 30 days apart, ($n_{1}=20,000$) or placebo ($n_{0}=10,000$). Enrollment occurred between August and September, 2020. Participants were followed in a blinded fashion until unblinding during March and April 2021. Open-label follow-up continued through the end of September 2021. Participants were assigned uniformly to one of 100 US-based sites, which were randomly distributed among the geographic regions for which the population model is available. The distribution of race and ethnicity reflected the demographics of the geographic locations of the sites, as reported by the US Census Bureau. Age was uniformly distributed between 18 and 85 and the prevalence of baseline pre-existing conditions were as follows: obesity in 25% of the population, chronic obstructive pulmonary disease (COPD) in 15%, diabetes in 25%, heart failure in 10%, neurological disorders in 10%, and immunosuppressive disorders in 7%. Covariate distributions were balanced in distribution across arms by virtue of randomization. Censoring due to study termination or outside-study COVID-19 vaccination was exponentially distributed and differential by age group, with a censoring rate of 0.5%/day for 18–64 and 1%/day for 65+ starting January 1, 2021. Daily risk of COVID-19 for each simulated individual was computed as described in the cohort model, and cases were generated using a Monte Carlo simulation. Sites tended to enroll individuals on average at 40% lower risk of SARS-CoV-2 infection than the local population at large $\left(\mu_{\alpha }=-0.5\approx ln(1-0.4),\sigma_{\alpha }=0.3\right)$. Incidence of SARS-CoV-2 and COVID-19 were 40% and 60% lower in the vaccine arm, respectively. The cohort model was used to simulate sero-positivity and study endpoints under these assumptions. Placebo arm follow-up is through censoring due to unblinding (Fig. 4A, blue curve) whereas vaccine arm follow-up is through end of study censoring (Fig. 4 green curve).

Based on the simulated participant characteristics and baseline sero-positivity, we estimate COVID-19 incidence throughout the entirety of primary follow-up using the cohort model (Fig. 4A, orange curve). The cohort model has wide uncertainty intervals to account for uncertainties in the site-level incidence, the impact of covariates such as age and race, and the differences between the study population and population at large. The estimated counterfactual placebo COVID-19 incidence curve (Fig. 4A, blue curve) preserves the shape of the cohort model but adjusts the incidence to match the observed placebo group incidence. In this example, the point estimates for the cohort model and counterfactual placebo COVID-19 incidence are quite close as the same assumptions were used to generate both the cohort model runs and the observed data. However the counterfactual placebo estimate is much more precise than the cohort model incidence estimate.

Monthly VE estimates are shown in Fig. 4B. We estimate observed VE until the crossover time and estimate counterfactual VE through all follow-up. The counterfactual VE estimate reasonably well estimates the true VE which is constant at 60%.

### 3.2. Reliable estimation of VE across multiple simulated trials under the modeling assumptions

**Fig. 5.**
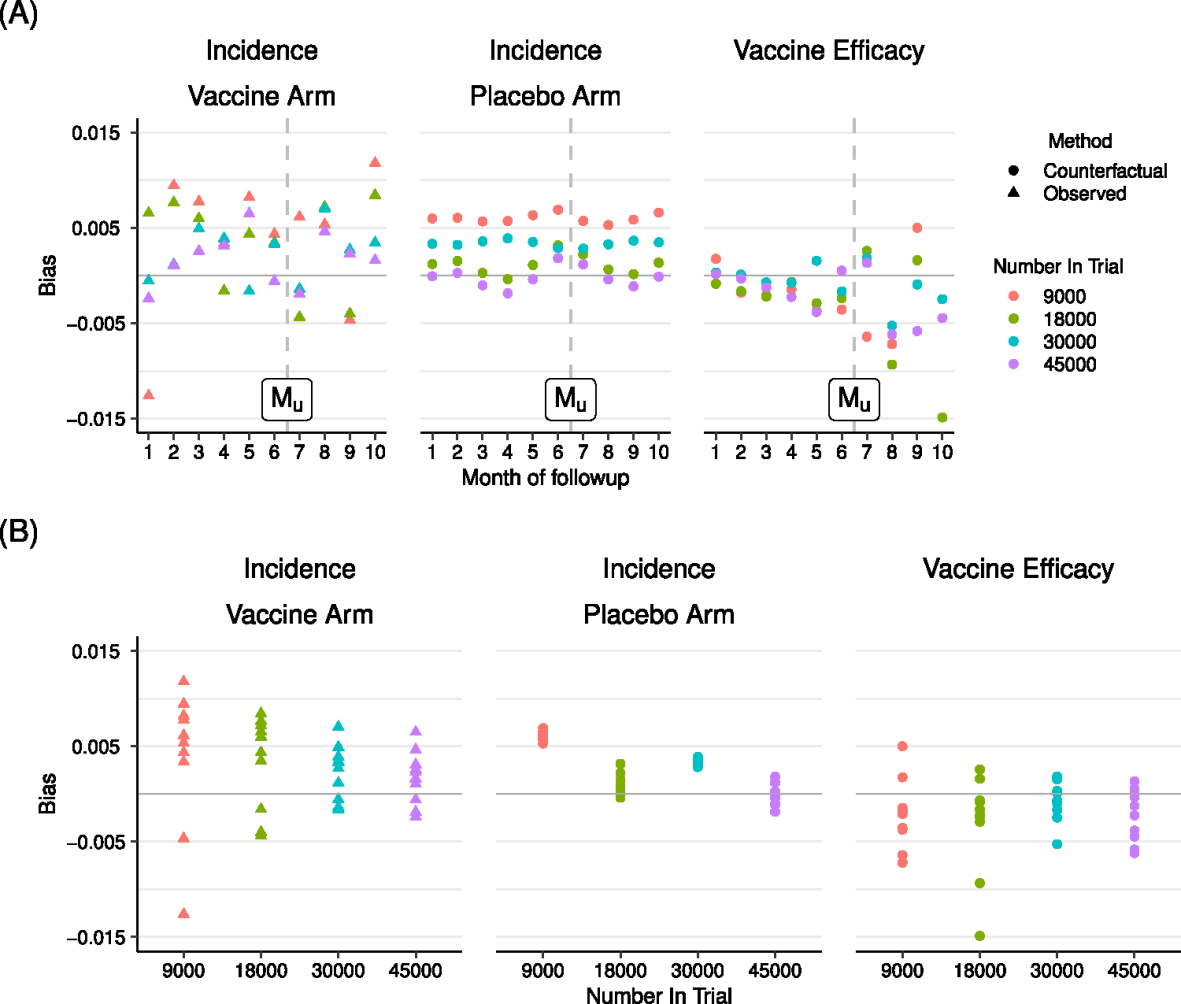
Relative bias of model estimates of COVID-19 incidence in the vaccine and placebo arms, and vaccine efficacy. Bias is plotted by month (A) and trial size (B). Observed vaccine arm incidence estimates, counterfactual placebo incidence estimates, and counterfactual vaccine efficacy estimates are shown. Horizontal line indicates the target bias of 0.00. Vertical dashed line indicates where the observed data is censored.

To evaluate whether our methodology provides unbiased estimates, ${\overset{ˆ}{\Lambda }}_{0}^{CF}(m)$ and ${\hat{VE}}_{CF}(m)$, of the true values, $\Lambda_{0}$ and VE(m) and accurately quantifies the uncertainty, we perform an extensive simulation study to evaluate the performance of our estimate across multiple simulated datasets and simulation scenarios. Using known values for the true parameters, we simulate data from a total of 4000 studies enrolling 9000, 18000, 30000, or 45000 participants and implement the methodology on the resulting data sets. The data-generating model for the trials is chosen to mimic features of USG-funded phase 3 COVID-19 VE trials, as described in Supplementary Materials Section S1.5. We first evaluate the relative bias of the monthly estimated counterfactual placebo COVID-19 incidence estimate, ${\overset{ˆ}{\Lambda }}_{0}^{CF}(m)$, and of the counterfactual VE estimate, ${\hat{VE}}_{CF}(m)$. We define relative bias as:

(13) $$Relative\;bias\;in\;placebo\;incidence=\frac{E_{N}[{\overset{ˆ}{\Lambda }}_{0}^{CF}]}{\Lambda_{0}(m)}-1$$

(14) $$Relative\;bias\;in\;Vaccine\;Efficacy=\frac{E_{N}[{\hat{VE}}_{CF}]}{VE(m)}-1$$

where $E_{N}$ represents an average across all simulated datasets with N enrollees. Relative bias in both incidence and VE estimates is less than 1% in most scenarios, with bias decreasing as the trial sample size increases (Fig. 5).

**Fig. 6.**
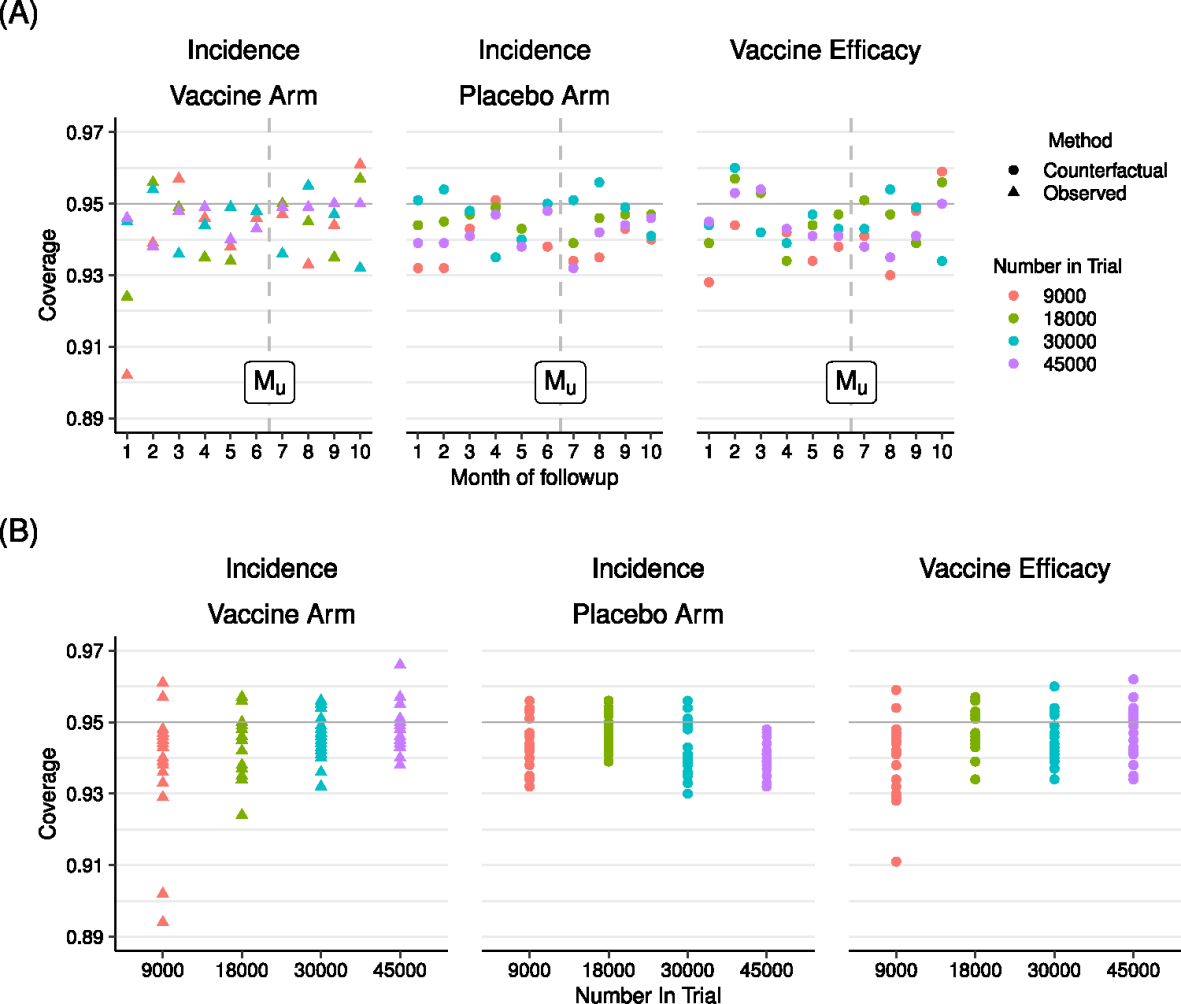
Coverage probability of nominal 95% uncertainty intervals of COVID-19 incidence in the vaccine and placebo arms, and VE. Coverage is plotted by month (A) and trial size (B). Observed vaccine arm incidence, counterfactual placebo incidence, and counterfactual vaccine efficacy are estimated. Horizontal line indicates the target coverage of 0.95. Vertical dashed line indicates the cross over month.

We also evaluate the coverage of the 95% uncertainty intervals, i.e the percentage of simulated datasets for which the calculated uncertainty interval contains the true value. Coverage is close to the nominal value of 95%, i.e. between 93% and 97%, across all scenarios (Fig. 6). The bias and coverage of our incidence estimates for the vaccine arm, based on observed data, are similar to those of the placebo arm, based on our counterfactual methodology.

### 3.3. Application: Estimation of VE over time in the phase 3 AZD1222 COVID-19 vaccine trial

Next we apply the methodology to data from the USG-funded phase 3 trial of the AZD1222 COVID-19 vaccine (ClinicalTrials.gov #NCT04516746), described in full elsewhere (Falsey et al., 2021; Sobieszczyk et al., 2022). Briefly, 32,380 individuals aged 18–101 in the US, Peru, and Chile at appreciable risk of COVID-19 who had no known prior SARS-CoV-2 infection were enrolled between August 28, 2020 and January 25, 2021. Participants were randomized in a 2:1 ratio to two doses of AZD1222 vaccine or placebo (saline) 28 days apart, and followed for incident COVID-19. Primary endpoint COVID-19 events were adjudicated events 14 or more days post second injection. Based on the primary analysis with a median of two months blinded follow-up, AZD1222 VE against COVID-19 was estimated as 74.0% (95% confidence interval (CI): 65.3 to 80.5) (Falsey et al., 2021). Participants were unblinded between November 25, 2020 and July 30, 2021. The unblinding before March 17, 2021 was by participant request, whereas the unblinding after this time was prompted by definitive evidence of vaccine efficacy having been established at the first interim efficacy analysis, which led to a subsequent decision to unblind all study participants. Many placebo recipients, and some AZD1222 recipients, chose to avail themselves of COVID-19 vaccines in the community; the median duration of blinded follow-up was 64 days post-second dose in the placebo group and 69 days post-second dose in the vaccine group. We restrict our analysis to US participants (89% of participants), given an inability to adequately calibrate population models for Peru and Chile. The crossover month, $M_{u}$, is March, 2021, at which time point 52% of placebo recipients remained under blinded follow-up and participants had been followed an average of approximately 50 days post-second dose. Placebo group follow-up was censored at unblinding and vaccine group follow-up was censored at the data cutoff of July 30, 2021.

**Fig. 7.**
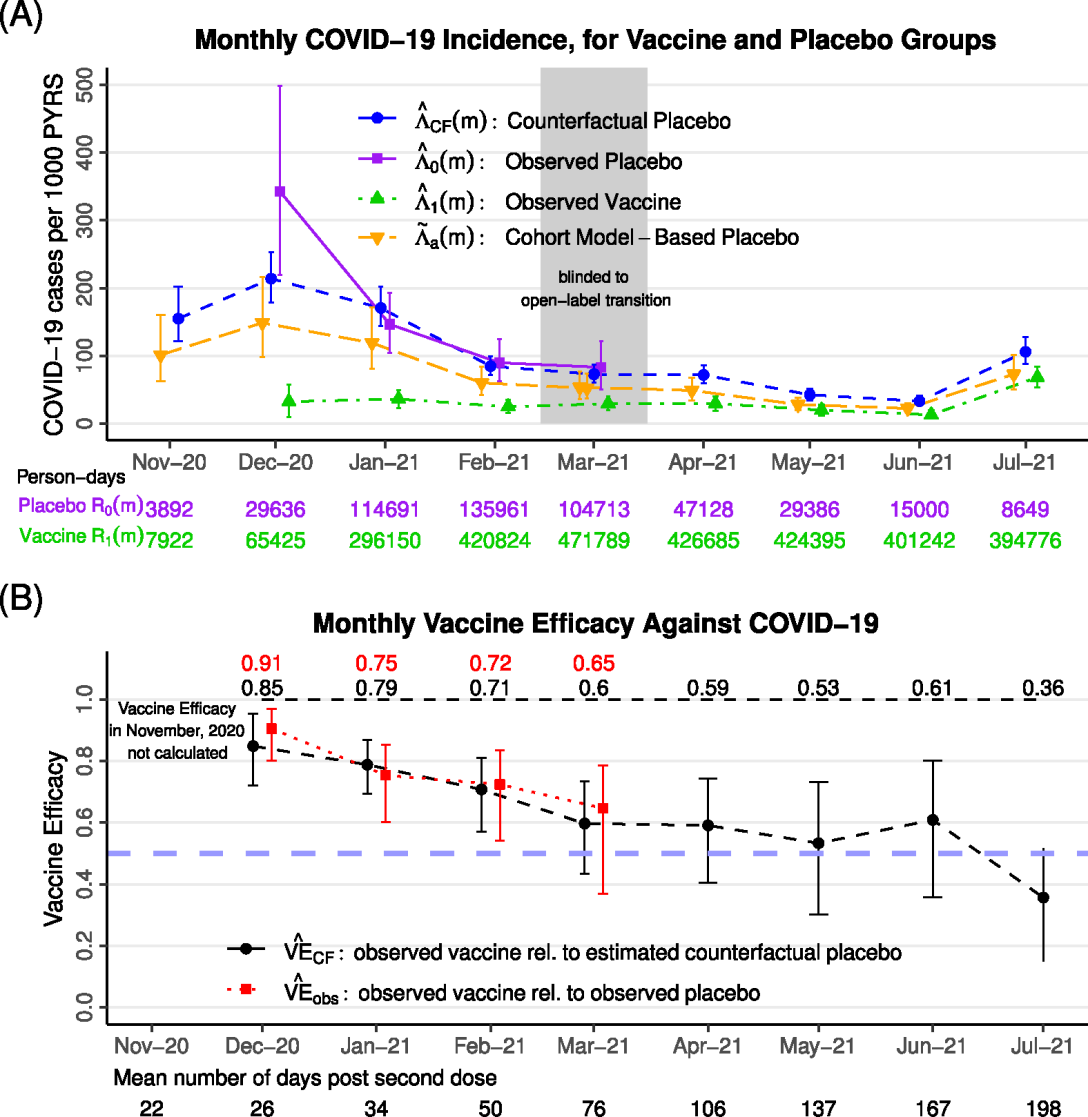
Estimated COVID-19 incidence for AZD1222 trial. (A) Observed vaccine and placebo group COVID-19 incidence is shown during blinded follow-up, and observed vaccine group incidence is shown during open-label follow-up. The cohort-model based estimate of incidence, and the estimate of counterfactual placebo incidence, is shown over all follow-up. (B) Estimated monthly VE, based on the contrast between observed vaccine vs. placebo group incidence during blinded follow-up (observed VE), and observed vaccine group vs. counterfactual placebo incidence during all follow-up (counterfactual VE). A reference line is shown at VE = 0.5.

Fig. 7A shows the estimated monthly observed COVID-19 incidence in the vaccine and placebo groups between December 2020 and March 2021. Incidence estimates in November 2020 are not shown given the small person-time available. The COVID-19 waves in the US in the winter of 2020–2021, and the beginning of the fall 2021 wave, are apparent.

Monthly AZD122 VE estimates are shown in Fig. 7B and tabulated in Supplementary Table S1. During blinded follow-up, observed VE estimates range from a high of 90.6% (95% uncertainty interval (UI): 80.1%–96.9%) in December, 2020 at a mean of 26 days post-second-dose to a low of 64.7% (95% UI: 36.9%–78.6%) in March, 2021 at a mean of 76 days post-second dose. These estimates are consistent with other analyses of the same data (Falsey et al., 2021; Sobieszczyk et al., 2022). During open-label follow-up, counterfactual VE estimates range between 36% and 61%. Counterfactual VE is estimated to be 59.1% (95% UI: 40.4%–74.3%) in April, 2021 at a mean of 106 days post-second-dose and falls to an estimated 35.7% (95% UI: 15.0%–51.7%) in July, 2021 at a mean of 198 days post-second-dose. The estimates are consistent with waning VE, although confidence intervals are wide and, importantly, remain well above the null.

AZD1222 VE estimates are qualitatively consistent with those of Sobieszczyk et al. (2022), who used an inverse-probability-weighting method to estimate an average VE of 61.7% (95% CI: 54.4%–67.8%) over both the blinded and open-label follow-up periods.

## 4. Discussion

This manuscript developed and implemented a methodology for estimating vaccine efficacy using counterfactual placebo COVID-19 incidence during open-label follow-up of a COVID-19 vaccine trial. We leveraged population surveillance data on diagnosed COVID-19, hospitalizations, and deaths to model COVID-19 incidence in a previously uninfected and unvaccinated population in the region around each trial site, and individual-level data on trial participants to adjust the incidence in the population to incidence in the trial cohort. We calibrated the model to the trial using COVID-19 incidence in the placebo arm during blinded follow-up. Our estimate of counterfactual placebo COVID-19 incidence for the open-label follow-up period accounts for uncertainty in the above modeling and estimation steps. We documented good performance of the methodology in simulations satisfying the model assumptions and applied it to estimate vaccine efficacy during open-label follow-up for the AZD1222 vaccine. We estimated AZD1222 vaccine efficacy to be 59.1% (95% UI: 40.4%–74.3%) in April, 2021 at a mean of 106 days post-second-dose and reduced to 35.7% (95% UI: 15.0%–51.7%) in July, 2021 at a mean of 198 days post-second-dose.

Other methods have been used to estimate COVID-19 vaccine efficacy during open-label follow-up (Sobieszczyk et al., 2022). In particular, inverse-probability-weighting (IPW) has been employed to leverage the available follow-up on placebo recipients who remained unvaccinated during the open-label period. The IPW method is compromised by the limited follow-up that remains on unvaccinated placebo recipients during open-label follow-up. Our methodology differs in that it leverages population SARS-CoV-2 and COVID-19 surveillance data external to the trial, and thus can provide more precise estimates of vaccine efficacy. However, we leverage complex mathematical models which incorporate numerous assumptions that are difficult to detail and interrogate; indeed, many assumptions are not testable using the data in hand. We view the two approaches as complementary and interestingly for the AZD122 vaccine trial the results of the two sets of analyses were consistent.

This methodology has application beyond the setting we considered. For example, it could be applied to other clinical endpoints influenced by vaccination, such as severe COVID-19. Severe COVID-19 was collected as a secondary endpoint in all phase 3 COVID-19 vaccine trials, thus, estimation of observed incidence and vaccine efficacy against this endpoint is straightforward. However, the cohort model would require modifications to account for different rates of severe disease by age, race, and comorbidities. Additionally, the methodology could be applied to estimating counterfactual placebo COVID-19 incidence for active-controlled or uncontrolled COVID-19 vaccine efficacy trials. The key challenge, however, would be the inability to calibrate the model to the trial population, given the absence of COVID-19 incidence for a veritable placebo arm during blinded follow-up. Finally, the methodological framework can be applied to other emerging pathogens where the rapid evaluation of a vaccine is required, necessitating early unblinding of trial participants.

The methodology has limitations that deserve consideration. First, our COVID-19 population model relies on assumptions about the extent of population-level immunity, which is in large part determined by the durability of immunity from vaccines available in the community. If the vaccine used in the trial was also being administered to the population at large, which was not the case in the US for the AZD1222 vaccine, then this would be problematic and one approach could be to fit the population model and regression model jointly. Second, population trends in COVID-19 incidence may vary by subpopulation within a given geographic region. Accounting for this would require stratification of the population model, which we did not do. Third, we leverage placebo-group COVID-19 incidence during blinded follow-up to estimate the offset between the model and the observed placebo incidence. We allow the offset to depend on geographic region and participant demographics, but assume that it is constant over time. This is a key assumption, assessed in our analysis but requiring careful assessment in future applications. Fourth, the methodology developed here estimates counterfactual placebo COVID-19 incidence, and vaccine efficacy against COVID-19, as a function of calendar time; time since last vaccination is an additional summary measure but not the time scale used. The methodology can potentially be extended to estimate VE on a study time scale by dividing the vaccine group into cohorts based on time of vaccination or by fitting a parametric model of efficacy as a function of study time. Fifth, the estimates of incidence and VE are subject to bias due to informative censoring or behavior changes that are not accounted for in the covariates used in the analysis.

Related to the assumption of a time-constant offset is the emergence of new SARS-CoV-2 variants over time. To the extent that there are different variants circulating before and after the crossover time, we assumed the offset estimated based on pre-crossover variants applied to post-crossover variants. Conceptually, our approach could be used to evaluate a variant-specific offset. Direct application would require sequencing the virus for all COVID-19 events. The population model would need to provide an estimate of variant-specific COVID-19 incidence in a naive population over time. This could be based on a combination of surveillance data on viral variants and modeling assumptions, using published studies describing the epidemiology and pathogenesis of different variants, including indirect vaccine effects on them. The main challenge would be the limited data available from the trial to estimate with reasonable precision the time-varying placebo-group COVID-19 incidence of any one variant during blinded follow-up. For the AZD1222 trial, Falsey et al. (2021) identified only 4 COVID-19 cases due to variants of concern during blinded follow-up. In addition, there were variants such as Delta and Omicron circulating post-crossover that did not exist pre-crossover, and so estimation of a variant-specific offset is not possible for this study. Different methodology is likely needed to estimate durability of variant-specific vaccine efficacy, see Follmann et al. (2022).

## Supplementary Material

1
