# Supplementary material for: Estimating vaccine efficacy during open-label follow-up of COVID-19 vaccine trials based on population-level surveillance data
Source: Epidemics. Author manuscript; Available in PMC 2024 Jul 18. (PMC11257040; doi:10.1016/j.epidem.2024.100768)
Supplement: 1 [file NIHMS2002861-supplement-1.pdf]

S1 Supplementary Material: Estimating Vaccine Efficacy During Open-Label Follow-up of COVID-19 Vaccine Trials Based on Population-level Surveillance Data

S1.1 Sensitivity analysis: censoring of placebo data

In our main analysis, we use all blinded follow-up data, however the time of unblinding of individual participants may be informative, e.g., participants who perceive themselves to be at higher COVID-19 risk may choose to be unblinded earlier [1]. To account for this potentially informative unblinding, we repeat the analysis, censoring all follow-up time at the end of the cross-over month M_u (Supplementary Figure S1). The impact of this change is to slightly increase both the counterfactual placebo incidence and vaccine efficacy, which is consistent with the hypothesis that individuals at higher risk will choose to become unblinded earlier (Supplementary Table S1). When censoring follow-up in this manner. The choice of M_u affects the bias and variance of the estimation. Selecting M_u as the last calendar month prior to the earliest unblinding of any placebo participant is one extreme. Another extreme is to select M_u as the last calendar month with any blinded primary follow-up person-time remaining in the placebo group. There is a tradeoff: a smaller M_u is expected to minimize bias due to informative unblinding, while a larger M_u is expected to maximize precision in estimating the offset. We repeat our analysis with a range of M_u from January, 2021 until May, 2021 to illustrate the potential sensitivity of the analysis to this parameter (Supplementary Table S2).

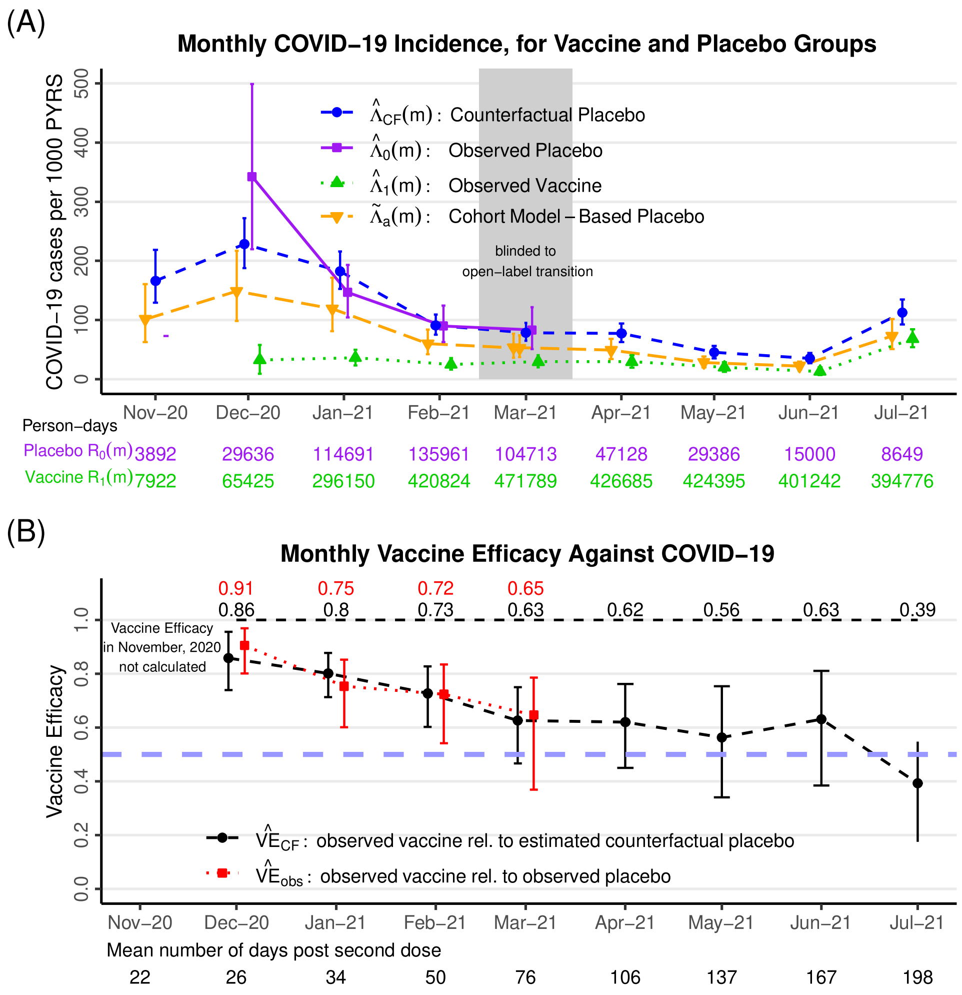

Figure S1: Estimated COVID-19 incidence for AZD1222 trial censoring all follow-up time after March 31, 2021. (A) Observed vaccine and placebo group COVID-19 incidence is shown during blinded follow-up, and observed vaccine group incidence is shown during open-label follow-up. The cohort-model based estimate of incidence, and the estimate of counterfactual placebo incidence, is shown over all follow-up. (B) Estimated monthly VE, based on the contrast between observed vaccine vs. placebo group incidence during blinded follow-up (observed VE), and observed vaccine group vs. counterfactual placebo incidence during all follow-up (counterfactual VE). A reference line is shown at VE = 0.5.

Table S1: Estimates of vaccine and placebo arm COVID-19 incidence and vaccine efficacy by month in the phase 3 trial of the AZD1222 COVID-19 vaccine. Observed vaccine and placebo arm incidence rates and observed vaccine efficacy estimates are shown for the blinded follow-up period, through March 2021. Counterfactual placebo incidence and vaccine efficacy estimates are shown over all follow-up. In the main analysis, we use all blinded follow-up censoring only at unblinding. We also show an alternative formulation, with censoring at the cross over month M_u. 95% uncertainty intervals accompany each point estimate.

	Vaccine Arm Incidence	Placebo Arm Incidence			Vaccine Efficacy		
	Observed	Observed	Counterfactual		Observed	Counterfactual	
Month			No Censoring (main)	Censoring at M_u		No Censoring (main)	Censoring at M_u
December, 2020	3.2 (0.9 - 5.7)	34.3 (22.0 - 49.9)	21.4 (18.1 - 25.8)	22.8 (18.8 - 27.2.x)	90.6 (80.1 - 96.9)	84.9 (72.1 - 95.7)	85.8 (73.9 - 95.7)
January, 2021	3.6 (2.3 - 5.0)	14.7 (10.4 - 19.3)	17.1 (14.4 - 20.2)	18.2 (15.3 - 21.6)	75.4 (60.2 - 85.2)	78.8 (69.4 - 88.9)	80.2 (71.3 - 87.8)
February, 2021	2.5 (1.6 - 3.5)	9.0 (6.2 - 12.4)	8.5 (7.1 - 10.0)	91 (7.5 - 10.9)	72.4 (54.1 - 83.4)	70.8 (57.1 - 81.1)	72.7 (60.3 - 82.8)
March, 2021	2.9 (2.0 - 4.0)	8.3 (5.1 - 12.2)	7.3 (6.0 - 8.7)	7.8 (6.5 - 9.5)	64.7 (36.9 - 78.6)	59.7 (43.4 - 73.4)	62.6 (46.6 - 75.1)
April, 2021	2.9 (1.9 - 4.0)	-	7.2 (5.9 - 8.6)	7.7 (6.3 - 9.4)	-	59.1 (40.4 - 74.3)	62.0 (44.9 - 76.2)
May, 2021	2.0 (1.2 - 2.8)	-	4.2 (3.4 - 5.2)	4.5 (3.5 - 5.6)	-	53.3 (30.2 - 73.1)	56.3 (34.1 - 75.4)
June, 2021	1.3 (0.7 - 2.0)	-	3.3 (2.6 - 4.1)	3.5 (2.7 - 4.4)	-	60.9 (35.7 - 80.2)	63.1 (38.4 - 81.1)
July, 2021	6.8 (5.4 - 8.4)	-	10.6 (8.9 - 12.7)	11.2 (9.3 - 13.5)	-	35.7 (15.0 - 51.7)	39.3 (17.5 - 54.8)

Table S2: Sensitivity of counterfactual estimates to the crossover month, M_u. In this sensitivity, we censor all trial participants at the end of the crossover month M_u and demonstrate the impact of varying M_u. 95% uncertainty intervals accompany each point estimate.

	Placebo Arm Incidence				Vaccine Efficacy			
	Cross-over month (M_u)				Cross-over month (M_u)			
Month	Jan, 2021	Feb, 2021	Apr, 2021	May, 2021	Jan, 2021	Feb, 2021	Apr, 2021	May, 2021
December, 2020	22.9 (18.1 - 28.3)	22.6 (18.2 - 27.4)	21.8 (18.1 - 26.0)	21.4 (17.8 - 25.5)	85.9 (73.2 - 95.2)	85.7 (73.1 - 95.4)	85.2 (72.7 - 95.9)	85.1 (72.8 - 95.4)
January, 2021	18.1 (14.4 - 22.5)	18.1 (14.8 - 21.5)	17.4 (14.6 - 20.6)	17.0 (14.4 - 20.3)	80.1 (69.9 - 88.2)	80.0 (70.7 - 87.9)	79.3 (69.8 - 87.2)	78.8 (69.3 - 86.9)
February, 2021	**9.1 (7.2 - 11.5)**	9.1 (7.4 - 11.0)	8.7 (7.3 - 10.3)	8.5 (7.1 - 10.0)	**72.8 (58.7 - 82.9)**	72.8 (60.0 - 82.6)	71.4 (57.9 - 81.7)	70.7 (56.8 - 81.2)
March, 2021	**7.9 (6.1 - 10.1)**	**7.9 (6.3 - 9.7)**	7.2 (6.0 - 8.5)	7.0 (5.8 - 8.3)	**62.8 (44.3 - 75.8)**	**62.9 (45.6 - 75.5)**	59.2 (42.1 - 72.8)	58.1 (40.6 - 72.2)
April, 2021	**7.7 (5.9 - 10.1)**	**7.7 (6.1 - 9.6)**	7.4 (6.1 - 8.8)	7.5 (6.2 - 9.1)	**61.7 (42.0 - 77.4)**	**61.8 (43.3 - 76.2)**	60.1 (41.9 - 75.1)	61.1 (43.0 - 75.6)
May, 2021	**4.4 (3.4 - 5.8)**	**4.5 (3.4 - 5.6)**	**4.3 (3.4 - 5.3)**	4.2 (3.4 - 5.2)	**55.3 (30.2 - 75.7)**	**55.4 (32.7 - 75.3)**	**54.2 (31.8 - 73.8)**	53.3 (30.7 - 73.3)
June, 2021	**3.4 (2.5 - 4.6)**	**3.4 (2.5 - 4.4)**	**3.4 (2.6 - 4.2)**	**3.3 (2.6 - 4.1)**	**62.0 (32.6 - 80.9)**	**62.1 (36.2 - 80.7)**	**61.5 (36.5 - 80.3)**	**60.7 (35.1 - 79.9)**
July, 2021	**10.9 (8.5 - 13.8)**	**10.9 (8.7 - 13.5)**	**10.8 (8.9 - 13.0)**	**10.5 (8.8 - 12.7)**	**37.3 (13.1 - 55.4)**	**37.4 (15.4 - 54.4)**	**36.7 (14.6 - 52.7)**	**35.2 (13.1 - 51.4)**

S1.2 Correcting for increasing immunity in the general population is crucial for accurate estimation of vaccine efficacy

Our methodology uses a model-based estimate of the SARS-CoV-2 incidence in a hypothetical naive population which has not been infected or vaccinated. This accounts for the growing immunity in the local population relative to those still at-risk in the counterfactual placebo arm, who have by definition not yet been infected or vaccinated. Without this adjustment, we would project a much lower incidence moving forward (Figure 2 green curves). We demonstrate the impact of this in our illustrative data set by generating a counterfactual placebo incidence using the estimated SARS-CoV2-infection in the local population rather than the hypothetical naive population (Supplementary Figure S3A). We replace equation (4) with the simplified version below.

$$\begin{aligned}
\text{Probability uninfected} \quad & P^{\text{Unf}}_{i,d} = \exp\left[-\gamma_{i,d}\right] P^{\text{Unf}}_{i,d-1} \qquad P^{\text{Unf}}_{i,E_i} = \nu_i \\
\text{Daily hazard rate of SARS-CoV-2} \quad & \gamma_{i,d} = \eta_{S_i,d} \\
\text{Daily endpoints} \quad & y_{i,d} = \left(1 - \exp\left[-\gamma_{i,d}\right]\right) P^{\text{Unf}}_{i,d-1} \qquad d > E_i \\
\text{Probability at risk} \quad & r_{i,d} = r_{i,d-1} - y_{i,d} \qquad r_{i,E_i} = 1
\end{aligned} \tag{S1}$$

Using this simplified approach, which doesn't account for changing population immunity, leads to an attenuated estimate of counterfactual placebo COVID-19 incidence (Figure S3A) and counterfactual VE: 32% by the end of the simulation, far lower than the true value of 60% (Figure S3B). Similarly, this naive approach also attenuates our estimates of counterfactual incidence and VE in AZD1222 (Figure S2). At the end of the open label follow-up period in July 2021, the counterfactual VE estimate is 15% (95% UI: -11% to 37%). The lower VE is due to the fact that incidence in the vaccine arm is now being compared to that of the general population, which was rapidly gaining immunity, rather than a true placebo. We observe a similar phenomenon in our simulated trial, with an estimated VE of only 36% at the end of open-label follow-up, much lower than the true value of 60% (Supplementary Figure S3).

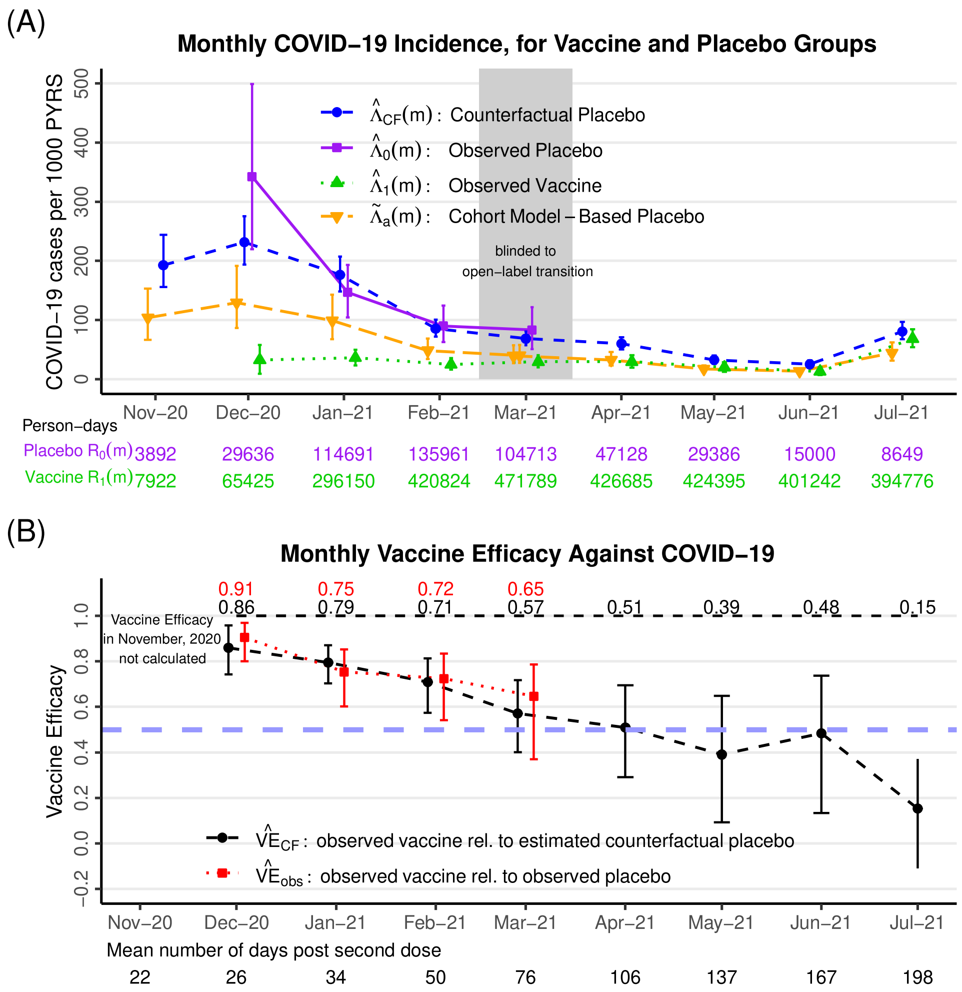

Figure S2: Estimated COVID-19 incidence for AZD1222 dataset based on a population model without adjustment for immunity in the population. (A) Estimated vaccine and placebo group COVID-19 incidence is shown during blinded follow-up, and vaccine group incidence is shown during open-label follow-up. A cohort-model based estimate of incidence and an estimate of counterfactual placebo incidence is shown over all follow-up. (B) Estimated monthly VE, based on the contrast between observed vaccine vs. placebo group incidence during blinded follow-up (observed VE), and observed vaccine group vs. counterfactual placebo incidence during all follow-up (counterfactual VE).

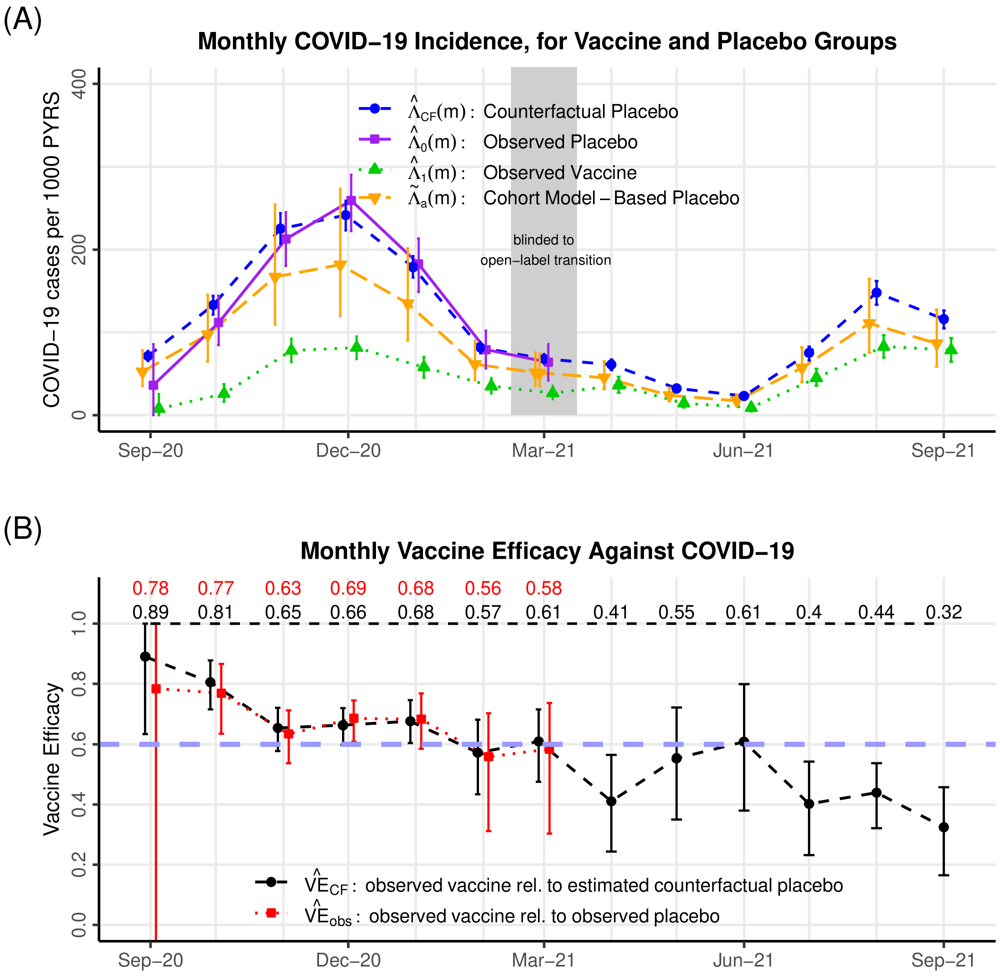

Figure S3: Estimated COVID-19 incidence for illustrative simulated dataset based on a population model without adjustment for immunity in the population. (A) Estimated vaccine and placebo group COVID-19 incidence is shown during blinded follow-up, and vaccine group incidence is shown during open-label follow-up. A cohort-model based estimate of incidence and an estimate of counterfactual placebo incidence is shown over all follow-up. (B) Estimated monthly VE, based on the contrast between observed vaccine vs. placebo group incidence during blinded follow-up (observed VE), and observed vaccine group vs. counterfactual placebo incidence during all follow-up (counterfactual VE).

S1.3 Cohort Model: Supplementary Methods

S1.3.1 SARS-CoV-2 Exposure Rates

As SARS-CoV-2 spreads through close contacts with infected individuals, risk of infection depends in part on factors such as the household size, school attendance, and place of employment. We divided exposure into two routes of infections 1) the participant is infected outside the home and 2) the participant is infected by a household member.

We used contact surveys from [2] to estimate how many contacts take place at home, work, school and in 'other' scenarios. We note both school children and working adults made an average of five contacts per day at school and work respectively. Children and young adults made an additional five 'other' contacts outside of the home, dropping to an average of three in middle age, and to two for the retired. We therefore set two contacts per day as a minimum 'essential' number of trips whereas additional contacts will relate to the amount of social interactions individuals have.

To calculate the participant's direct risk of SARS-CoV-2 infection outside the home, NHAR_0, we estimate the number of daily contacts. Each contact has a risk-per-contact of infection, RPC. RPC varies dramatically in time and space as the prevalence of SARS-CoV-2 fluctuates, as described by the population model, so in the exposure model we focus solely on the number of contacts. Participants who went into their school/workplace once, two to four times, or five times a week have on average N_{Work} = 1, 3, or 5 contacts daily, respectively. A participant employed in a job considered anything beyond 'low-risk' by OSHA has an additional $N_{\mathrm{OSHA}} = 5$ contacts per day. A participant who has, within the last month, attended a gathering of 1-10, 11-20, 21-50, 51 to 250, or 250+ had an additional $N_{\mathrm{Gathering}}$ = 1, 2, 3, 4, or 5 daily contacts, respectively. Finally participants were assumed to have two daily 'essential' contacts as a baseline.

$$\mathrm{NHAR}_0 = (2 + N_{\mathrm{Work}} + N_{\mathrm{OSHA}} + N_{\mathrm{Gathering}})\mathrm{RPC} \tag{S2}$$

The risk of SARS-CoV-2 infection via a household member, HAR, depends on the number of people in the participant's household under age 18 ($N_{<18}$), ages 18-64 (N_{18-64}), and over age 65 ($N_{\geq 65}$). For each age group, we made an assumption about their risk of being infected outside of the home, $\mathrm{NHAR}_{\mathrm{age}}$. We assume that household members under the age of 18 had on average five contacts outside of the home, due to socializing, as schools were generally closed during the 2020-2021 school year or following social distancing protocols. In addition, their risk-per-contact is assumed to be 50% less than the participants' [3]. Household members ages 18-64 also have

on average five contacts outside of the home — three due to socializing and two more due ongoing employment. Household members age 65+ had on average two 'essential' contacts outside. The total risk of infection from household sources, HAR, is the sum of NHAR over all household members multiplied by the secondary attack rate, SAR = 20% [4].

$$\begin{gathered}\mathrm{HAR} = \mathrm{SAR}\left(N_{<18}\mathrm{NHAR}_{<18} + N_{18-64}\mathrm{NHAR}_{18-64} + N_{\geq 65}\mathrm{NHAR}_{\geq 65}\right) \\ \mathrm{NHAR}_{<18} = 2.5 * \mathrm{RPC} \quad \mathrm{NHAR}_{18-64} = 5 * \mathrm{RPC} \quad \mathrm{NHAR}_{\geq 65} = 2 * \mathrm{RPC}\end{gathered} \tag{S3}$$

The total SARS-CoV-2 infection rate was HAR + NHAR_0. After normalizing by RPC, we derive an "exposure score".

$$\text{Exposure Score} \qquad \mathrm{ES} = 2 + N_{\mathrm{Work}} + N_{\mathrm{OSHA}} + N_{\mathrm{Gathering}} + 0.5 * N_{<18} + N_{18-64} + 0.4 * N_{\geq 65} \tag{S4}$$

This exposure score represents the effective number of non-household contacts an individual made each day either directly or through secondary transmission via a household member. Using data from the CoVPN screening registry [5, 6], we estimated average exposure score by age and race, $\mathrm{ES}_{0,i}$ (Table S3). We impute exposure score using participants' age and race if one or more of the underlying components is missing for a participant. If either age or race are missing, we use the average exposure score from each site, using census data.

$$\beta^{\alpha} V_i = \begin{cases} \ln\left(\mathrm{ES}_i\right) & \text{All behavioral risk score variables available} \\ \ln\left(\mathrm{ES}_{0,i}\right) & \text{At least one behavioral risk score missing, demographic information available} \\ \ln\left(\mathbb{E}^{S_i}\left[\mathrm{ES}_{0,i}\right]\right) & \text{Otherwise} \end{cases} \tag{S5}$$

S1.3.2 Risk Model of COVID-19

We estimate the individual risk of COVID-19, conditional on SARS-CoV-2 infection, using individual data on age and comorbidities.

$$\beta^{\rho} V_i = \beta^{\rho}_0 + \beta^{\rho}_{\mathrm{age}} \mathrm{age}_i + \sum_{\mathrm{comorbidity}} \beta^{\rho}_{\mathrm{comorbidity}} I_{\mathrm{comorbidity}} \tag{S6}$$

Where age_i is subject age in years and $I_{\mathrm{comorbidity}}$ is an indicator function for a specific comorbidity. We derive the intercept, $\beta^{\rho}_0 = -1.57 \pm 0.20$, and age dependence, $\beta^{indsymp}_{\mathrm{age}} = 0.030 \pm 0.004 \mathrm{years}^{-1}$, by fitting a linear model to values from a prior modeling study [3] (Supplemental Figure S4, blue curve). We use estimates of $\beta^{\rho}_{\mathrm{comorbidity}}$ from a study of medicare data [7] (Supplemental Table S4).

Table S3: Average exposure score by age and race, $ES_{0,i}$, representing the expected number of daily non-household contacts either directly or via a household member.

Age Group	American Indian/ Alaskan Native	Asian/ Pacific Islander	Black/ African American	Hispanic/ Latino	Mixed	Other	White	Overall
18-19	9.27	7.07	7.98	7.90	8.90	8.15	8.81	8.55
20-24	8.17	6.98	8.43	8.99	8.08	8.40	8.26	8.19
25-29	8.54	6.79	7.76	8.61	7.41	8.50	7.24	7.37
30-34	9.42	7.27	8.02	8.59	7.43	7.61	7.29	7.43
35-39	9.29	7.43	7.97	8.44	7.52	8.31	7.58	7.66
40-44	7.97	7.65	8.05	8.73	7.39	8.50	7.66	7.77
45-49	8.15	7.82	7.93	9.00	7.57	9.02	7.70	7.87
50-54	8.44	7.74	7.66	8.74	7.22	7.77	7.41	7.58
55-59	6.98	7.35	7.06	8.25	6.93	7.64	6.86	7.02
60-64	6.85	6.35	6.66	7.23	5.87	6.51	5.85	5.98
65-69	5.12	5.16	4.82	5.59	4.76	5.10	4.51	4.58
70-74	3.61	4.30	4.03	4.79	4.04	4.59	3.71	3.76
75-79	3.79	4.34	4.22	4.07	4.24	5.94	3.46	3.53
80-84	3.75	4.05	5.49	4.21	4.27	3.60	3.46	3.52
85-89	3.91	3.32	2.85	4.82	3.13	2.34	3.60	3.59
90-94	N/A	2.94	2.87	4.70	3.26	4.38	4.00	3.96
Overall	7.17	7.07	7.07	8.14	7.04	7.48	6.27	

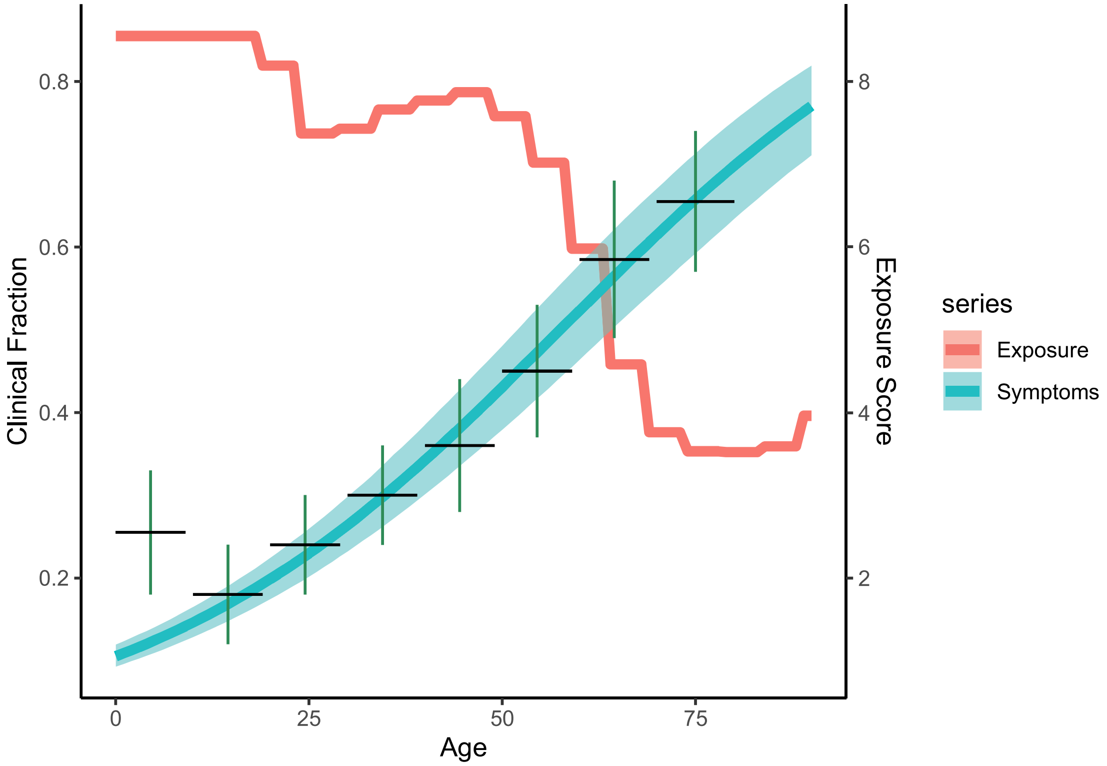

Figure S4: Risk of SARS-CoV-2 exposure (red line) and COVID-19 (blue curve) by age. Exposure risk is based on the marginal average in the CoVPN registry (Table S3), risk of symptoms are interpolated based on a logistic fit to data from [3] (segments). Note that the fit is only applicable to individuals eligible to enroll in the trial, i.e. 18 years and older.

Table S4: Prevalence and associated risk of COVID-19 from the CoVPN risk score [7]

	Age 18-64		Age >65	
Comorbidity	%	$\beta^{\rho}_{\text{comorbidity}}$	%	$\beta^{\rho}_{\text{comorbidity}}$
Obesity	27.7	0.21 (0.18 – 0.24)	19.7	0.10 (0.09 – 0.12)
Currently Smoking	24.2	-0.28 (-0.31 – -0.24)	6.8	-0.03 (-0.05 – -0.01)
A history of smoking or vaping	8.2	-0.07 (-0.11 – -0.03)	13.8	0.07 (0.05 – 0.08)
Asthma	8.3	0.26 (0.22 – 0.31)	5.0	0.20 (0.18 – 0.23)
COPD	13.3	0.31 (0.27 – 0.35)	12.0	0.37 (0.36 – 0.39)
Diabetes (Type 1)	0.2	0.27 (0.01 – 0.54)	0.2	0.02 (-0.09 – 0.14)
Diabetes (Type 2)	22.9	0.24 (0.21 – 0.27)	24.7	0.22 (0.21 – 0.23)
Pulmonary Fibrosis	0.7	0.19 (0.08 – 0.30)	1.2	0.11 (0.07 – 0.15)
Cystic Fibrosis	0.1	0.69 (0.42 – 0.96)	0.0	-0.13 (-0.68 – 0.41)
Heart Arrythmia	5.0	0.08 (0.04 – 0.13)	15.6	0.05 (0.04 – 0.07)
Cardiomyopathy	2.6	-0.03 (-0.09 – 0.04)	3.2	-0.04 (-0.07 – -0.02)
Heart Disease	12.3	0.26 (0.22 – 0.29)	21.8	0.05 (0.03 – 0.06)
Coronary Artery Disease	3.9	-0.03 (-0.08 – 0.02)	6.2	0.02 (0.00 – 0.03)
Hypertension	46.4	0.12 (0.09 – 0.15)	67.4	0.15 (0.14 – 0.17)
Congestive Heart Failure	7.5	0.15 (0.10 – 0.20)	11.8	0.22 (0.20 – 0.24)
Stroke, TIA, or cerebrovascular disease	3.7	-0.02 (-0.08 – 0.03)	3.7	0.10 (0.08 – 0.12)
Neurological Conditions	13.4	0.55 (0.52 – 0.58)	8.8	0.78 (0.76 – 0.79)
Immunocompromising or Immunosuppressive Condition	9.7	0.33 (0.29 – 0.36)	6.2	0.31 (0.30 – 0.33)
Inflammatory Bowel Disease	1.4	0.03 (-0.07 – 0.13)	1.1	0.08 (0.04 – 0.13)
Multiple Sclerosis	1.8	-0.08 (-0.17 – 0.02)	0.3	0.21 (0.14 – 0.29)
Arthritis (Rheumatoid)	3.2	0.06 (0.00 – 0.13)	2.5	0.11 (0.08 – 0.14)
Other Autoimmune Disease	7.3	0.04 (-0.01 – 0.09)	5.9	0.06 (0.03 – 0.08)
Kidney Disease	10.6	0.19 (0.15 – 0.23)	17.4	0.13 (0.11 – 0.14)
Liver Disease	2.8	0.19 (0.13 – 0.24)	1.2	0.17 (0.14 – 0.21)
Sickle Cell Anemia	0.3	0.04 (-0.12 – 0.19)	0.0	0.04 (0.13 – 0.20)

S1.3.3 Calculation of positivity at baseline

Given an individual's risk of infection and disease, we calculated the probability that they test positive at the time of enrollment due to a prior or ongoing infection. All enrolled individuals fall into three categories defined by an infection status which we index with the variable IS

1. They were never infected, i.e they are naive. (IS=Nai)
2. They were asymptomatically infected and never confirmed.(IS=Asy)
3. They were symptomatically infected and never confirmed. In this instance, they must not be currently symptomatic to meet the enrollment criteria. (IS=Unr)

Let $Q_i^{\text{IS},\pm}$ be the probability that individual i in each of these three circumstances returned a positive or negative test, respectively. The probability of being positive at baseline is the probability of testing positive divided by the probability of enrolling. The probability of being naive given test-negativity is the probability of being naive divided by the total probability of being test-negative.

$$\begin{aligned}\xi_i &= \frac{Q_i^{\text{Nai+}} + Q_i^{\text{Unc+}} + Q_i^{\text{Asy+}}}{Q_i^{\text{Nai+}} + Q_i^{\text{Unc+}} + Q_i^{\text{Asy+}} + Q_i^{\text{Nai-}} + Q_i^{\text{Unc-}} + Q_i^{\text{Asy-}}} \\ v_i &= \frac{Q_i^{\text{Nai-}}}{Q_i^{\text{Nai-}} + Q_i^{\text{Unc-}} + Q_i^{\text{Asy-}}}\end{aligned} \tag{S7}$$

The probability of an unenrolled individual remaining naive on calendar date d, $P_{i,d}^{\text{Unf}}$, iss calculated iteratively. Individuals arere assumed to be uninfected on date d_0 chosen to be Jan 1st, 2020.

$$\begin{aligned}P_{i,d}^{\text{Unf}} &= \exp\left[-\gamma_{i,d}\right] P_{i,d-1}^{\text{Unf}} \\ P_{i,d_0}^{\text{Unf}} &= 1\end{aligned} \tag{S8}$$

We assume that there are no false positives, therefore the probability of being naive at baseline and testing positive is zero.

$$\begin{aligned}Q_i^{\text{Nai+}} &= 0 \\ Q_i^{\text{Nai-}} &= P_{i,E_i}^{\text{Unf}}\end{aligned} \tag{S9}$$

The probability of an individual with unconfirmed SARS-CoV-2 infection on any day $d \leq E_i$ enrolling and testing positive (or negative) is the product of 1) probability of infection on that day of the infection rate on that day, $\left(P_{i,d}^{\text{Unf}} - P_{i,d-1}^{\text{Unf}}\right)$, 2) the probability of an infection being unconfirmed and being asymptomatic/symptomatic $F_{S_i,d}^{\text{Asy/Sym}}$, and 3) the probability that a person infected on day d will be positive/negative at enrollment $C_{E_i-d}^{\text{IS},+/-}$ (see §S1.3.5). The total probability of individuals enrolling with prior infection is obtained by summing across

all pre-enrollment days. We assume that prior to enrollment, symptomatic infections are much more likely to be detected than asymptomatic. Specifically, we assume that asymptomatic cases are only detected if all symptomatic cases have been detected and therefore any further increase in detection rates must be among asymptomatic infections.

$$
\begin{aligned}
F_{S_i,d}^{Asy} &= \begin{cases} 1-\rho_i & \rho_i \geq \frac{\delta_{S_i,]}}{\eta_{S_i,d}} \\ 1-\frac{\delta_{S_i,d}}{\eta_{S_i,d}} & \rho_i < \frac{\delta_{S_i,d]}}{\eta_{S_i,d}} \end{cases} \\
F_{S_i,d}^{Sym} &= \begin{cases} \rho_i-\frac{\delta_{S_i,d}}{\eta_{S_i,d}} & \rho_i \geq \frac{\delta_{S_i,d}}{\eta_{S_i,d}} \\ 0 & \rho_i < \frac{\delta_{S_i,d]}}{\eta_{S_i,d}} \end{cases} \\
Q_i^{\text{Asy}\pm} &= \sum_{d=d_0}^{E_i} C_{E_i-d}^{\text{Asy},+/-} F_{S_i,d}^{Asy} \left(P_{i,d}^{\text{Unf}} - P_{i,d-1}^{\text{Unf}}\right) \\
Q_i^{\text{Unc}\pm} &= \sum_{d=d_0}^{E_i} C_{E_i-d}^{\text{Unc},+/-} F_{S_i,d}^{Sym} \left(P_{i,d}^{\text{Unf}} - P_{i,d-1}^{\text{Unf}}\right)
\end{aligned}
\tag{S10}
$$

S1.3.4 Calibration of model

As described in the previous section, we estimate the probability of being positive at baseline for each individual, ξ_i. These probabilities depend non-linearly on the individual relative susceptibilities, α_i. They also depend on the site-specific adjustment term α_S which accounts for any differences in SARS-CoV-2 exposure between the enrolled trial cohort at each study site and the general population in the study site's location.

We approximate the posterior distribution of α_S using stochastic approximation of expectation-maximization (Kuhn, Laveille). In this iterative procedure, both the study-level parameters, μ_α and σ_α, and the site-level α_{S_i} are alternately estimated. During each step of the iteration, we:

1. Perform k_1 steps of markov-chain monte carlo optimization of α_S. Each proposed step in the chain is generated by sampling the site-level parameters from the global-study level distribution based on the current estimate of the population parameters. Proposed steps are evaluated by computing the site-specific likelihood

$$
L_S = \underbrace{\prod_{i|S_i=S} \xi_i^{B_i}(1-\xi_i)^{1-B_i}}_{\text{Likelihood of Baseline status}} \tag{S11}
$$

   where ξ_i are recalculated at each step. Following (Kuhn, Laveille), $k_1 = 5$.

2. Recalculate the global study-level parameters based on the current estimates of α_S. Then return to step 1 and continued the markov chains for another k_1 steps. We repeated the entire procedure $k_2 = 20$ times.

Our iterative calibration procedure requires an initial guess for the population variables μ_α and σ_α. We obtain this initial estimate using the baseline SARS-CoV-2 status, ξ_i and taylor expansion to create a local, logistic approximation to this quantity.

$$\begin{aligned}
\text{logit}\xi_i &= \text{logit}\xi_i|_{\alpha_{S_i}=1} + \frac{\partial \text{logit}\xi_i}{\partial \alpha}|_{\alpha_{S_i}=1}\left(\alpha_{S_i}-1\right) + o\left(\left(\alpha_{S_i}-1\right)^2\right) \\
&\simeq \underbrace{\text{logit}\xi_i|_{\alpha_{S_i}=1} - 1}_{\text{offset}=f_i} + \alpha_{S_i} \\
&= f_i + \alpha_{S_i}
\end{aligned} \tag{S12}$$

We used logistic regression model with a mixed effect $\alpha_{S_i} \sim N(\mu_\alpha, \sigma_\alpha)$ and offset f_i to calculate an initial estimate of μ_α and σ_α.

S1.3.5 Modeling sero-positivity post-infection

The the probability of being positive or negative at baseline t days after SARS-CoV-2, $C_t^{\text{IS,+/-}}$, was derived from a simple pharmacokinetic model. We assumed infected individuals seroconverted at a rate $k_1 = 0.1\text{days}^{-1}$ and that they seroreverted at a rate k_2^{IS} which was drawn from a beta-distributed prior for each cohort run derived from clinical data.[8]

$$\begin{aligned}
&\text{Infected} & \frac{d\text{I}^{\text{IS}}}{dt} &= -k_1 \text{I}^{\text{IS}} \\
&\text{Seropositive} & \frac{d\text{SP}^{\text{IS}}}{dt} &= k_1 \text{I}^{\text{IS}} - k_2^{\text{IS}} \text{SP}^{\text{IS}} \\
&\text{Seronegative} & \frac{d\text{SN}^{\text{IS}}}{dt} &= k_2^{\text{IS}} \text{SP}^{\text{IS}}
\end{aligned} \tag{S13}$$

IS	$C_t^{\text{IS,+}}$	$C_t^{\text{IS,-}}$	k_2^{IS} Prior
Unc	$\text{SP}^{\text{Unc}}(t)$	$\text{SN}^{\text{Unc}}(t)$	$\ln(\beta(27,4))/56$
Asy	$\text{I}^{\text{Asy}}(t) + \text{SP}^{\text{Asy}}(t)$	$\text{SN}^{\text{Asy}}(t)$	$\ln(\beta(18,12))/56$

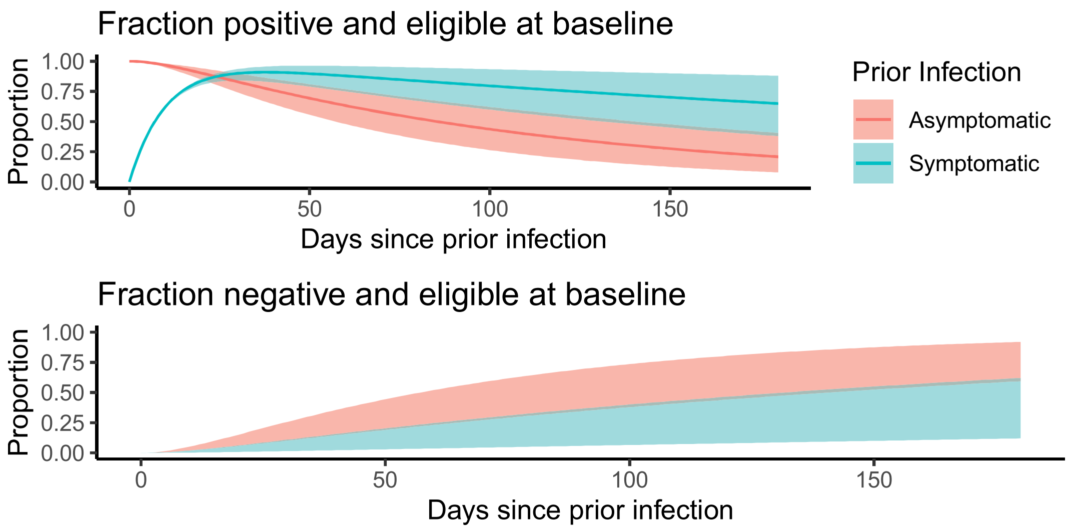

Figure S5: Probability of an trial participant being trial eligible (asymptomatic) and either positive or negative at enrollment t days after infection. Upper panel probability of being positive following symptomatic, $C_t^{\text{Unc},+}$, or asymptomatic infection, $C_t^{\text{Asy},+}$. Lower panel: probability of being negative following symptomatic, $C_t^{\text{Unc},-}$, or asymptomatic infection, $C_t^{\text{Asy},-}$. Note that for individuals with symptomatic infection, probabilities do not sum to one as those who are currently symptomatic are not eligible.

S1.4 Assessing changes to offset during blinded follow-up

A key assumption of our methodology is that the offset, μX, remain constant in time. Although the validity of this assumption cannot be tested during the open-label extension, we can test whether or not it remains constant during blinded follow-up. To quantify the change in we augment equation 6, with a time-varying term μ_m:

$$\log E\left(Y_0^X(m)\right) = \log R_0^X(m) + \log \tilde{\Lambda}_{0,a}^X(m) + \mu X + \mu_m. \tag{S14}$$

To summarize the offset across all strata, we use the incidence rate ratio between the counterfactual model and cohort model during blinded follow-up.

$$\text{Incidence ratio} = \frac{\sum_X \hat{w}_z^x(m)\hat{\Lambda}_0^{CF,x}(m)}{\sum_X \hat{w}_z^x(m)\tilde{\Lambda}_{0,a}^x(m)} \tag{S15}$$

For our application, we computed the incidence ratio during the blinded follow-up of the AZD1222 COVID vaccine trial using both a time-constant offset model (equation 6 and Figure S6 green circles) and a time-varying offset (equation S14 and Figure S6 purple triangles). The uncertainty intervals of the ratio computed with a time-varying offset overlap with those computed using a time-constant offset. This means that there is no evidence of a time-varying offset during baseline follow-up and provides some support to our assumption.

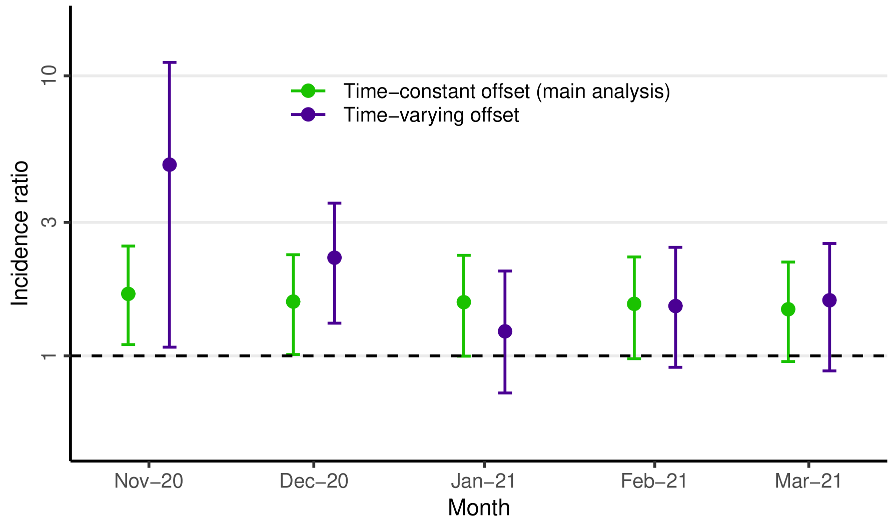

Figure S6: Ratio of marginal counterfactual placebo incidence estimate to cohort model incidence using a time constant offset model (green circle) or time-varying offset model (purple triangle). Dots = point estimates, error bars = 95% uncertainty interval.

S1.5 Bias and Coverage Estimates

S1.5.1 Simulation Setting

We simulate a vaccine trial that extends over M months, with M_1 months prior to crossover and the remaining $M - M_1$ months open-label follow-up. We assume a piecewise-constant true COVID-19 incidence, $\Lambda_{\zeta}^{x}(m)$, that varies by month (m) and covariates $X = (X_1, X_2)$. To mimic the successive waves of COVID-19 incidence, we assume the true incidence follows a log-transformed sinusoid with median baseline value $\zeta_1 = 0.001$ infections per person-month, an initial log-scale amplitude $\zeta_2 = 1$ corresponding to seven-fold difference between peaks and troughs, and frequency $\zeta_3 = 0.5$ per month. Over the course of the trial the magnitude of oscillations decreases by ζ_4 and the oscillation was modified by covariate x_1 by a factor ζ_5. To mimic differences in peak timing in different regions of the US, the frequency is modulated by the covariate X_1, which corresponded to geographic region, via a coefficient ζ_5. Both X_1 and X_2, which corresponds to demographic group, also affects the the magnitude of incidence: individually by factors ψ^{x_1} and ϕ^{x_2}, and together by the interaction term $\gamma\psi^{x_1}\phi^{x_2}$. These parameters result in $15\% - 18\%$ cumulative incidence over the 6 month open-label follow-up, similar to the real-world trial outcome. Incidence in the vaccine arm is multiplied by a factor $1 - VE(m)$. We assume $VE(m)$ starts at 90% in

month $m = 1$ and drops linearly to 40% by month $m = M$.

$$\log \Lambda_z^{x=(x_1,x_2)}(m) = f(m, x_1) + \psi^{x_1} + \phi^{x_2} + \gamma \psi^{x_1} \phi^{x_2} + \log(1 - \varepsilon_z(m))$$
$$f(m, x_1) = \log(\zeta_1) + \left(\zeta_2 - \zeta_4 \frac{m-1}{M}\right) \sin\Big((\zeta_3 + \zeta_5 x_1)(m-1)\Big) \tag{S16}$$
$$\varepsilon_0 = 0 \qquad \varepsilon_1(m) = \mathrm{VE}_1 \frac{M-m}{M-1} + \mathrm{VE}_2 \frac{m-1}{M-1}$$

For the first M_1 months, we assume an annual censoring rate that varies by stratum of $c^X = c_0 + c_1 X_1 + c_1 X_2$. All remaining participants are censored at the end of month M_1.

S1.5.2 Simulation of Survival Data

We simulate 1000 trials of N individuals for each of N=9000, 18000, 30000, or 45000. For all trials, the ratio of vaccine to placebo arm participants is 2:1 and the number in each covariate stratum is fixed according to the proportions f_{X_1} and f_{X_2}. In each simulation the individual trial endpoints $Y_0^X(m)$ and follow-up time $R_0^X(m)$ are stochastically generated using a monte-carlo algorithm.

1. For each individual, two poisson distributed random numbers with rate one, p_{inc} and p_{cens} are generated.

2. The time of censoring (in months) is calculated assuming a constant hazard rate of censoring with an annual cumulative censoring rate of c^X or at the end of follow-up, whichever came first. The end of follow-up is month M_1 for placebo arm participants and M for vaccine arm participants.

$$T_{\text{cens}} = \begin{cases} \min\left\{\frac{12 p_{\text{cens}}}{\log(1-c^X)}, M_1\right\} & \text{Participant in placebo arm} \\ \min\left\{\frac{12 p_{\text{cens}}}{\log(1-c^X)}, M\right\} & \text{Participant in vaccine arm} \end{cases} \tag{S17}$$

3. The month of a study endpoint, m_{inc}, is the first month where the the cumulative hazard of infection, $C_z^x(m)$ exceeds p_{inc}. In the case that the cumulative hazard never exceeds p_{inc} then there is no endpoint.

$$C_z^x(m) = \sum_{m'=1}^{m} \Lambda_z^x(m')$$
$$m_{\text{cens}} = \min\left\{m | C_z^x(m) > \log(1 - p_{\text{cens}})\right\} \tag{S18}$$

4. If there is a study endpoint, we calculate the time of infection (in months) using the assumption of piecewise constant incidence. If there is no endpoint, then $T_{\text{inc}} = \infty$.

$$T_{\text{inc}} = \begin{cases} m_{\text{cens}} - \frac{C_z^x(m_{\text{cens}}) - p_{\text{cens}}}{\Lambda_z^x(m_{\text{cens}})} & \text{Infection during follow-up} \\ \infty & \text{No infection during follow-up} \end{cases} \tag{S19}$$

5. Finally, we define T_{end} to be the minimum of T_{cens} and T_{inc}. There is a study endpoint only if $T_{\text{inc}} \leq T_{\text{cens}}$

We also simulate cohort model estimates of disease incidence. Following (6), we assume that the cohort model incidence, $\tilde{\Lambda}^{x}_{0,\text{coh},j}(m)$ for $j = 1, \ldots, 100$ differs from the true incidence $\log \Lambda_z^{x=(x_1,x_2)}(m)$ by a linear offset that depends on X_1, X_2 and cohort run j, but not the calendar month m. This offset is generate for each cohort run by sampling values $\psi^{X_1}_{\text{coh},j}$ and $\phi^{X_1}_{\text{coh},j}$.

$$
\begin{aligned}
\log \tilde{\Lambda}^{x}_{0,p,j}(m) &= f(m, x_1) + \psi^{x_1} + \phi^{x_2} + \gamma_{\text{coh}} \psi^{x_1} \phi^{x_2} \\
\psi^{X_1}_{\text{coh},j} &\sim \mathcal{N}(\mu_{\text{coh},\psi}, \sigma_{\text{coh},\psi}) \\
\phi^{X_1}_{\text{coh},j} &\sim \mathcal{N}(\mu_{\text{coh},\phi}, \sigma_{\text{coh},\phi})
\end{aligned}
\tag{S20}
$$

Table S5: Simulation parameters

Parameter	Description	Value
ζ_1	Baseline incidence	0.001
ζ_2	Incidence oscillation amplitude	1
ζ_3	Incidence oscillation frequency	0.5
ζ_4	Modulation of incidence oscillation amplitude by m	0.5
ζ_5	Modulation of incidence oscillation frequency by X_1	0.5
ψ^{x_1}	True log-scale effect of X_1 relative to baseline	1.5, 2.0, 2.5, 3.0
ϕ_{x_2}	True log-scale effect of X_2 relative to baseline	0.0, 0.3, 0.2, 0.6
γ_0	True interaction between X_1 and X_2	-0.1
$\mu_{\text{coh},\psi}$	Mean log-hazard by X_1 in cohort model	1.0, 0.5, 1.0, 0.0
$\sigma_{\text{coh},\psi}$	Standard deviation of log-hazard by X_1 in cohort model	0.25, 0.5, 0.1, 0.3
$\mu_{\text{coh},\phi}$	Mean log-hazard by X_2 in cohort model	0.0, 0.3, 0.2, 0.6
$\sigma_{\text{coh},\phi}$	Standard deviation of log-hazard by X_2 in cohort model	0
γ_{coh}	Interaction between X_1 and X_2 in cohort model	-0.1
M	Number of months follow-up	10
M_1	Number of months of blinded follow-up	6
f_{X_1}	Fraction of participants by X_1	0.1, 0.2, 0.3, 0.4
f_{X_2}	Fraction of participants by X_2	0.1, 0.2, 0.3, 0.4
c_0	Base annual censoring	10%
c_1	Stratum specific censoring	10%
VE_1	Initial vaccine efficacy	90%
VE_2	Final vaccine efficacy	40%

References

[1] Tsiatis AA, Davidian M. Estimating vaccine efficacy over time after a randomized study is unblinded. Biometrics. 2021 Aug. Available from: https://doi.org/10.1111/biom.13509.

[2] Prem K, Cook AR, Jit M. Projecting social contact matrices in 152 countries using contact surveys and demographic data. PLoS computational biology. 2017;13(9):e1005697.

[3] Davies NG, Klepac P, Liu Y, Prem K, Jit M, Eggo RM. Age-dependent effects in the transmission and control of COVID-19 epidemics. Nature medicine. 2020;26(8):1205-11.

[4] Jing QL, Liu MJ, Zhang ZB, Fang LQ, Yuan J, Zhang AR, et al. Household secondary attack rate of COVID-19 and associated determinants in Guangzhou, China: a retrospective cohort study. The Lancet Infectious Diseases. 2020;20(10):1141-50.

[5] Lora AJM, Long JE, Huang Y, Baden LR, Sahly HME, Follmann D, et al. Rapid Development of an Integrated Network Infrastructure to Conduct Phase 3 COVID-19 Vaccine Trials. JAMA Network Open. 2023 Jan;6(1):e2251974. Available from: https://doi.org/10.1001/jamanetworkopen.2022.51974.

[6] Network CP. Together we can stay ahead of COVID-19;. https://www.coronaviruspreventionnetwork.org/.

[7] Siford S, Chillarige Y, Avagyan A, Sung HM, Li Y, Smith E, et al. CoVPN Risk Score Specifications; 2020. Unpublished (Acumen LLC).

[8] Long QX, Tang XJ, Shi QL, Li Q, Deng HJ, Yuan J, et al. Clinical and immunological assessment of asymptomatic SARS-CoV-2 infections. Nature medicine. 2020;26(8):1200-4.
